# Dynamic Electron Redistribution on Rooted-Type High-Entropy Perovskite-Supported Ir Catalysts for Durable Proton-Exchange-Membrane Water Electrolysis

**DOI:** 10.1021/jacs.6c13780

**Published:** 2026-08-29

**Authors:** Jing Ni, Yuan Wang, Zhaoping Shi, Cheng Huang, Mingrui Yu, Heng Liu, Meiling Xiao, Changpeng Liu, Hao Li, Wei Xing

**Affiliations:** † Hydrogen Energy Industry Institute of Jilin Province, Changchun Institute of Applied Chemistry, 58277 Chinese Academy of Sciences, Changchun 130022, China; ‡ School of Applied Chemistry and Engineering, University of Science and Technology of China, Hefei 230026, China; § Advanced Institute for Materials Research (WPI-AIMR), 13101 Tohoku University, Sendai 980-8577, Japan; ∥ CIAC - HKUST Joint Laboratory for Hydrogen Energy, Changchun 130022, China

## Abstract

Addressing the inherent trade-off between low iridium loading and operational durability is of paramount importance for advancing proton-exchange-membrane water electrolysis (PEMWE) technology toward green hydrogen production, yet it remains a formidable challenge. Here, we develop a multilevel stabilization strategy to dynamically stabilize Ir under industrial current densities. This approach combines strong physical anchoring, achieved by constructing a rooted-type embedded architecture on a high-entropy perovskite support (Ir/HESIO), with dynamic electronic state regulation of the active supported layer via in situ electron redistribution. In situ spectroscopic characterization and theoretical simulations jointly verify that the synergy between structural confinement and in situ electronic regulation effectively suppresses the migration, overoxidation, and subsequent dissolution of Ir active species. Owing to these attributes, the developed Ir/HESIO exhibits a low cell voltage of 1.71 and 1.80 V to achieve industrial current densities of 2 and 3 A cm^–2^, meeting the DOE 2026 target (1.80 V @ 3A cm^–2^) and outperforming most reported catalysts. Moreover, it demonstrates outstanding operational stability, maintaining stable operation over 3800 h at 2 A cm^–2^ with just 2.2% potential decay and over 3200 h at 3 A cm^–2^ with just 4.7% potential decay. This work not only presents a highly promising electrocatalyst for water electrolysis but also establishes a dynamic electronic structure regulation strategy that is broadly applicable for stabilizing other heterogeneous catalysts.

## Graphical abstract

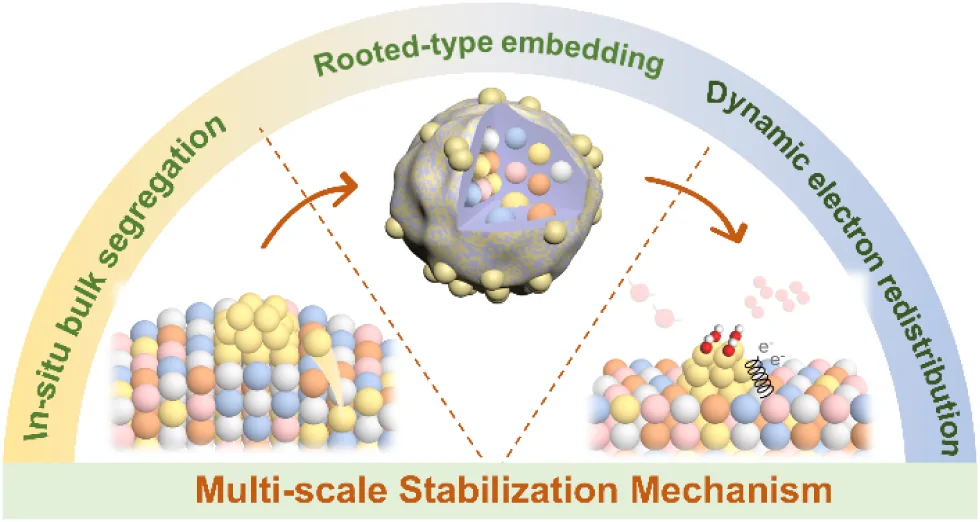

## Introduction

The pursuit of a sustainable future necessitates a paradigm shift in the global energy structure. Hydrogen, as a green and zero-emission energy carrier, is poised to play a pivotal role in the forthcoming energy landscape. Proton-exchange-membrane water electrolysis (PEMWE) represents a crucial technology for converting renewable electricity into clean and high-purity hydrogen, offering a sustainable pathway to address energy and environmental challenges.^1–3^ A central bottleneck, however, resides at the anode side, where the oxygen evolution reaction (OER) proceeds. As a complex four-proton-coupled electron transfer process, the OER operates under harshly acidic and oxidizing conditions, imposing stringent demands on catalyst performance.^4,5^ Although iridium-based materials currently represent the state-of-the-art anode catalysts for practical PEMWE systems,^6–9^ their widespread deployment is severely limited by the extreme scarcity and high cost of iridium.^10–12^ This drives an urgent need to drastically reduce iridium usage in practical applications while preserving or even enhancing catalytic activity and, most critically, ensuring long-term durability under industrial operating conditions.^1,13^ Achieving this balance between ultralow iridium usage and robust stability remains a formidable challenge.

The operational instability of low-iridium OER catalysts stems from several interconnected degradation pathways. Primarily, the high anodic potentials required for OER can induce excessive adsorption and overoxidation dissolution of iridium sites.^14–16^ Concurrently, metastable low-valent species (e.g., Ir^3+^), which often serve as precatalytic centers, are susceptible to direct dissolution.^17,18^ Furthermore, participation of lattice oxygen in the reaction pathway can lead to the formation of oxygen vacancies, triggering gradual structural collapse of the catalyst bulk.^19,20^ Critically, these pathways are not independent; for instance, lattice oxygen oxidation can accelerate the direct dissolution of adjacent low-valent iridium and even induce severe oxidative dissolution.^21–23^ This interplay results in a complex, multimechanistic deactivation process. Compounding this challenge, the active sites undergo continuous dynamic restructuring, including frequent adsorption/desorption of oxygen intermediates (e.g., O*, OH*, and OOH*)^24^ and rapid shifts in the local electronic environment, making the stabilization of low-iridium catalysts particularly formidable.

To address these intertwined degradation pathways, a multiscale stabilization strategy is highly required. At the atomic level, it necessitates precise electronic modulation on iridium sites to intrinsically enhance their antioxidation ability under high anodic potentials. At the micronanoscale, well-defined geometric confinement is required to suppress the migration, aggregation, and dissolution of iridium species during operation. High-entropy perovskite oxides provide an ideal material platform to realize this concept: their stable configuration^25^ and tunable coordination environments^26^ enable dynamic regulation of the electronic states of iridium centers, while their segregation can generate geometrically confined, ultrafine Ir nanoclusters within a stable oxide matrix. This dual capability, simultaneous electronic and structural stabilization, positions high-entropy perovskite oxides as a highly promising direction^27^ for developing high-performance, low-iridium anode catalysts.

Inspired by this understanding, we developed a novel multiscale stabilization paradigm based on a rooted catalyst architecture, where iridium active sites are dynamically stabilized by a designed high-entropy perovskite (HESIO) substrate. Through controlled bulk phase segregation, we achieved the high-temperature in situ construction of an integrated Ir/HESIO catalyst. Advanced characterization confirms a rooted structure, with uniformly dispersed iridium in the surface-enriched active layer embedded on the perovskite bulk, establishing a structural basis for continuous dynamic regulation. In situ X-ray absorption spectroscopy and theoretical simulations reveal that the HESIO substrate dynamically modulates the electronic environment of surface Ir sites during operation, suppressing overcoordination and progressive oxidation-key pathways for Ir dissolution. The strong embedding of the active sites, coupled with the dynamic electron redistribution, furnishes a robust structural and electronic foundation for sustained OER activity. The resulting Ir/HESIO anode exhibits exceptional stability in the PEMWE single cell, showing potential degradation of only 2.2% over 3800 h at 2 A cm^–2^ and 4.7% over 3200 h at 3 A cm^–2^, respectively. This work shows a new paradigm that integrates dynamic electronic modulation with interfacial space confinement, addressing the stringent stability-loading requirements for practical PEMWE anode catalysts.

## Results and Discussion

### Design and Structural Characterization of Rooted-Type Catalysts

We conceived a multiscale stabilization strategy that combines physical anchoring and dynamic electronic regulation for low-iridium-loading, stable OER catalysts (Figure 1). In contrast to the conventional approach of external Ir loading, we constructed a rooted-type architecture via in situ Ir segregation, which enables effective embedding of Ir clusters. Our strategy involves the introduction of both stable (e.g., Ti and Mo) and active (e.g., Co and Ni) B-site elements^28^ into the perovskite lattice to form a highly heterogeneous, Ir-containing high-entropy perovskite. The pre-experiment of single-element doping Ir/SrIr­(M)­O_3_ (M = Ni, Mo, Co, Ti) profoundly elucidates the indispensable role of incorporated elements within the expected high-entropy configuration (Figure S1): Ti and Mo provide the essential structural rigidity to maintain the perovskite framework, while reducible elements such as Ni and Co offer the necessary possibilities for supported-layer modulation, collectively achieving the optimal synergy between structural robustness and catalytic activity. Through selective Ir segregation, the rooted-type supported catalyst is constructed while preserving the perovskite host and forming new Ir crystal phases. Ir is thereby distributed within both the perovskite bulk and the supported active surface layer, providing structural integrity and dynamic stability. On one hand, the Ir clusters are stabilized by strong embedding during their formation from the bulk phase. On the other hand, the Ir coordination and local electric environment can be in situ regulated under operating conditions by leveraging the excellent electronic flexibility of high-entropy perovskite. Therefore, the stabilization of the catalyst’s bulk structure and its adaptability under reaction conditions establish a robust foundation for its practical application.

**Figure 1:**
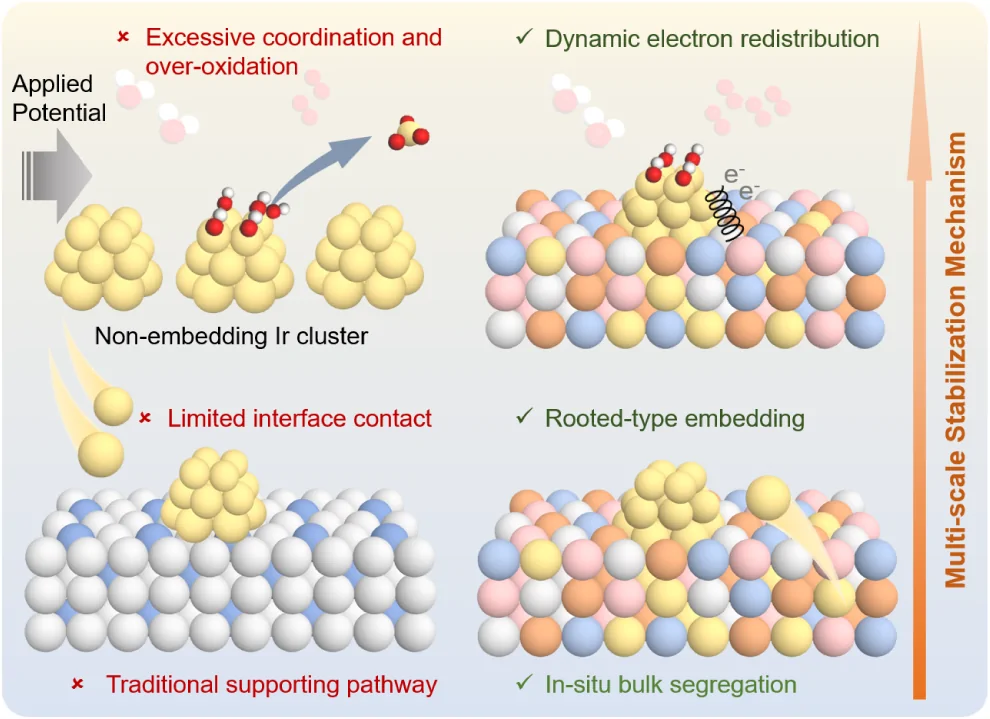
Design scheme of rooted-type supported catalysts.

The rooted-type supported catalyst was synthesized through post-treatment of an Ir-containing high-entropy perovskite (Figure 2a). First, a high-entropy perovskite oxide precursor, SrIr­(TiMoCoNi)­O_3_ (denoted as HESIO), was prepared using a sol–gel method.^29,30^ Subsequently, the target catalyst was in situ fabricated through an exsolution treatment of the HESIO precursor at a high temperature under a reducing atmosphere.^31^ Because of the different affinities^32,33^ of the B-site-doped elements with H_2_, the precious metal Ir preferentially segregates onto the surface to form a supported catalyst (Ir/HESIO). There may be partial cosegregation of Ni and Co elements, but the metallic Ni and Co are dissolved during the subsequent acid-etching process. Therefore, this method finally leverages the partial exsolution of B-site Ir cations from the perovskite lattice to generate well-dispersed Ir nanoparticles that are firmly embedded on the parent oxide surface. For comparison, the unsupported iridium catalyst (denoted as Ir) was synthesized by using a similar method but without additional B-site cation doping.

**Figure 2:**
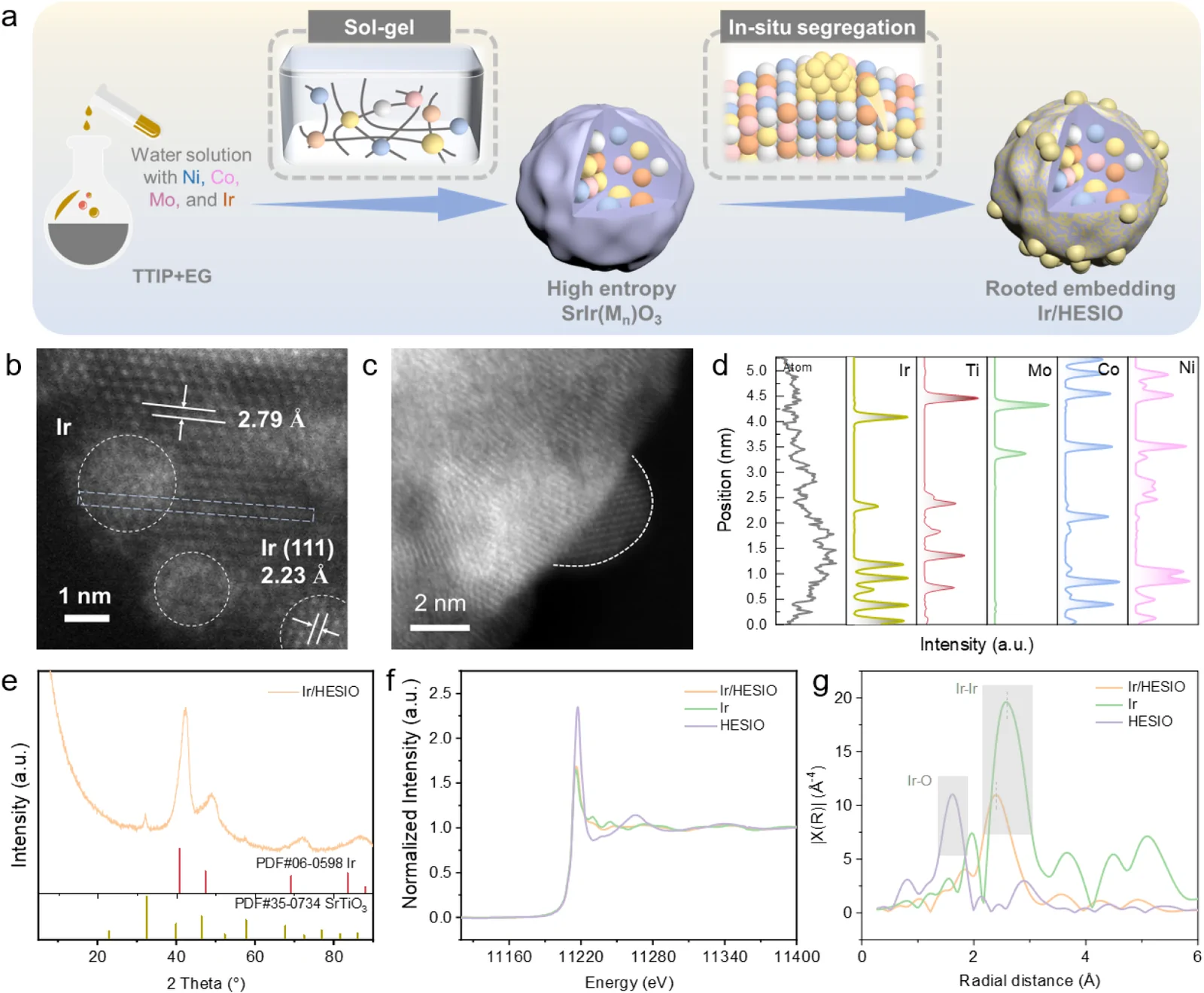
(a) The scheme of the synthesis method for Ir/HESIO. (b) The atomic-resolution HAADF-STEM image of Ir/HESIO. (c) The HAADF-STEM image of the interface for Ir/HESIO. (d) The atomic-resolution EDS element profile of Ir/HESIO, shows atomic Ir, Ti, Mo, Co, and Ni intensity line profiles in the selected area of panel b. (e) The XRD pattern of Ir/HESIO. (f) The XANES spectroscopy and (g) The EXAFS spectroscopy of as-prepared catalysts.

The morphology and microstructure of the Ir/HESIO catalyst were directly examined by transmission electron microscopy (TEM). The atomic-resolution high-angle annular dark-field scanning transmission electron microscopy (HAADF-STEM) image of the support region (Figure S2a) reveals a lattice spacing of 2.8 Å, corresponding to the cubic perovskite (110) interplanar spacing. The high-density and small-sized nanoparticles are uniformly and continuously dispersed on the catalyst surface, exhibiting clear lattice features characteristic of exsolved Ir clusters (Figure S2b–c). Furthermore, the HAADF-STEM image of Ir/HESIO simultaneously captures the lattice fringes of both the HESIO support and the supported Ir nanoparticle (Figure 2b). The HAADF-STEM image (Figure 2c, Figure S3) of the interface between Ir nanoparticles and the HESIO support directly evidences the embedding structure, demonstrating their intimate interfacial contact. The TEM images (Figure S4) of the unsupported Ir sample reveal a stacked morphology consisting of nonuniform nanoparticles, demonstrating the antiagglomeration effect of the high-entropy HESIO support on the high-temperature in situ growth of Ir nanoparticles in Ir/HESIO catalyst. The element distribution within the catalyst was investigated by energy-dispersive spectroscopy (EDS) mapping and line-scan analysis.^34–36^ The EDS line-scan profile of the HESIO precursor (Figure S5) demonstrates a homogeneous distribution of all five B-site dopant elements in the bulk phase. The EDS mapping and atomic-resolved elemental profile (Figure S6) also confirm the uniform dispersion of the B-site elements at the atomic level in the support region. These results collectively indicate the successful formation of an atomically well-mixed high-entropy support. A cross-catalyst EDS line scan (Figure S7) was further performed on the Ir/HESIO catalysts, revealing significant surface enrichment of Ir while a partial Ir signal persists within the support bulk. This observation is strongly corroborated by the high Ir intensity observed in the line profile of the near-surface cluster region (Figure 2d). The localized Electron Energy Loss Spectroscopy (EELS) analysis was further performed, focusing on the support region, which clearly exhibits a distinct Ir M-edge signal at approximately 2040 eV alongside the O K-edge signal at about 533 eV (Figure S8). This simultaneous detection of Ir and O signals in the bulk region confirms the partial maintenance of Ir within the perovskite lattice. Therefore, the Ir cations migrated from the perovskite lattice during high-temperature reduction, subsequently nucleating and growing into surface-anchored clusters. Meanwhile, the incomplete segregation of iridium atoms, induced by the sluggish diffusion effect of the high-entropy support,^37^ promotes the formation of the anchoring structure of the rooted type.

X-ray diffraction (XRD) was conducted to determine the crystalline phases of the as-prepared catalysts. The XRD pattern (Figure 2e) of Ir/HESIO displays distinct diffraction peaks corresponding to both the perovskite phase (JCPDS No.35-0734) and metallic Ir phase (JCPDS No.06-0598). The high intensity of the metallic Ir peak confirms the crystalline nature of the exsolved Ir nanoparticles, corresponding to the pronounced lattice fringes observed in HAADF-STEM images. The above results confirm the composite structure of Ir nanoparticles supported on high-entropy perovskites. In contrast, the XRD patterns of the HESIO precursor and the unsupported Ir catalyst exhibit only the single perovskite phase or the single metallic Ir crystal phase, respectively (Figure S9), underscoring the unique composite structure of Ir/HESIO achieved via the segregation process. Furthermore, the atomic ratios of different B-site elements were quantified by inductively coupled plasma optical emission spectrometry (ICP-OES, Table S1). The Ir content in Ir/HESIO is higher than that in untreated HESIO, a difference attributed to the dissolution of unstable metallic B-site elements that cosegregated with Ir during acid washing. Rietveld refinement of XRD patterns^38^ (Figure S10) indicates that the metallic Ir phase constitutes approximately 45 wt % of the Ir/HESIO catalyst. On the basis of the ICP-OES results, the remaining Ir content within the HESIO support is 13 wt %. Therefore, the atomic ratio of B-site metals in the Ir/HESIO support can be determined (Table S2), allowing the molecular formula of the composite to be identified as 45 wt % Ir/Sr_0.16_(Co_0.29_Ir_0.24_Ti_0.21_Mo_0.04_Ni_0.22_)­O_3_. The moderate Ir mass fraction is beneficial to its performance expression in the PEMWE membrane electrode.^39,40^

To probe the local coordination environment and electronic state of Ir at the atomic level, we conducted X-ray absorption near-edge structure (XANES) and extended X-ray absorption fine structure (EXAFS) spectroscopy at the Ir L_3_-edge.^41,42^ The XANES spectra of Ir/HESIO and the Ir catalyst exhibit significantly lower white-line peak intensity and a negative energy shift compared with the HESIO precursor (Figure 2f, Figure S11a). This indicates the reduction of Ir to a low-valent metallic state near metallic Ir (Figure S11b), consistent with the XRD patterns. In the Fourier-transformed EXAFS spectra in R space (Figure 2g), the local coordination environment of Ir/HESIO is dominated by a strong Ir–Ir scattering path, in contrast to the Ir–O coordination characteristic of the HESIO support. This confirms the successful extraction of Ir from the oxygen-coordinated lattice environment to form metallic Ir–Ir bonds. Notably, the Ir–Ir bond distance in Ir/HESIO is shorter than that in the Ir catalyst , originating from the lattice compression caused by transient cosegregation of active B-site elements and subsequent selective dissolution, as well as a modulating effect of the HESIO support on the supported Ir catalytic layer.

The above characterization elucidates the key structural features of the designed Ir/HESIO catalyst: (i) homogeneous, atom-scale mixing of B-site elements in the HESIO support; (ii) Ir nanoclusters formed, then uniformly and continuously dispersed on the HESIO support surface with intimate contact via an in situ segregation process; and (iii) rooted-type structure construction with iridium distributed both on the surface catalytic layer and the perovskite support, with the element content in each phase quantified. These innovative microstructural attributes establish a robust foundation for the manifestation of the catalytic performance.

### Electrochemical Performance in a Three-Electrode System

The intrinsic catalytic activity and operational stability of the prepared catalysts were rigorously assessed in a standard three-electrode system using 0.5 M H_2_SO_4_ as the electrolyte. Linear sweep voltammetry (LSV) was first performed to evaluate OER activity. As shown in the polarization curves (Figure 3a), the Ir/HESIO exhibits the highest performance, with an overpotential of only 273 mV to achieve a current density of 10 mA cm^–2^, which is significantly lower than those of HESIO (340 mV) and the Ir counterpart (327 mV) by 67 mV and 54 mV, respectively. To exclude the influence of catalyst loading on the electrode, mass-specific activities were calculated to highlight the intrinsic efficiency of the active sites (Figure S12). The Ir/HESIO catalyst delivers a remarkable mass activity of 285 A g^–1^ at a potential of 1.58 V, outperforming HESIO and Ir references by 4.2 and 6.8 times, respectively. The outstanding intrinsic activity demonstrates the maximized noble-metal utilization enabled by the rooted-type architecture.

**Figure 3:**
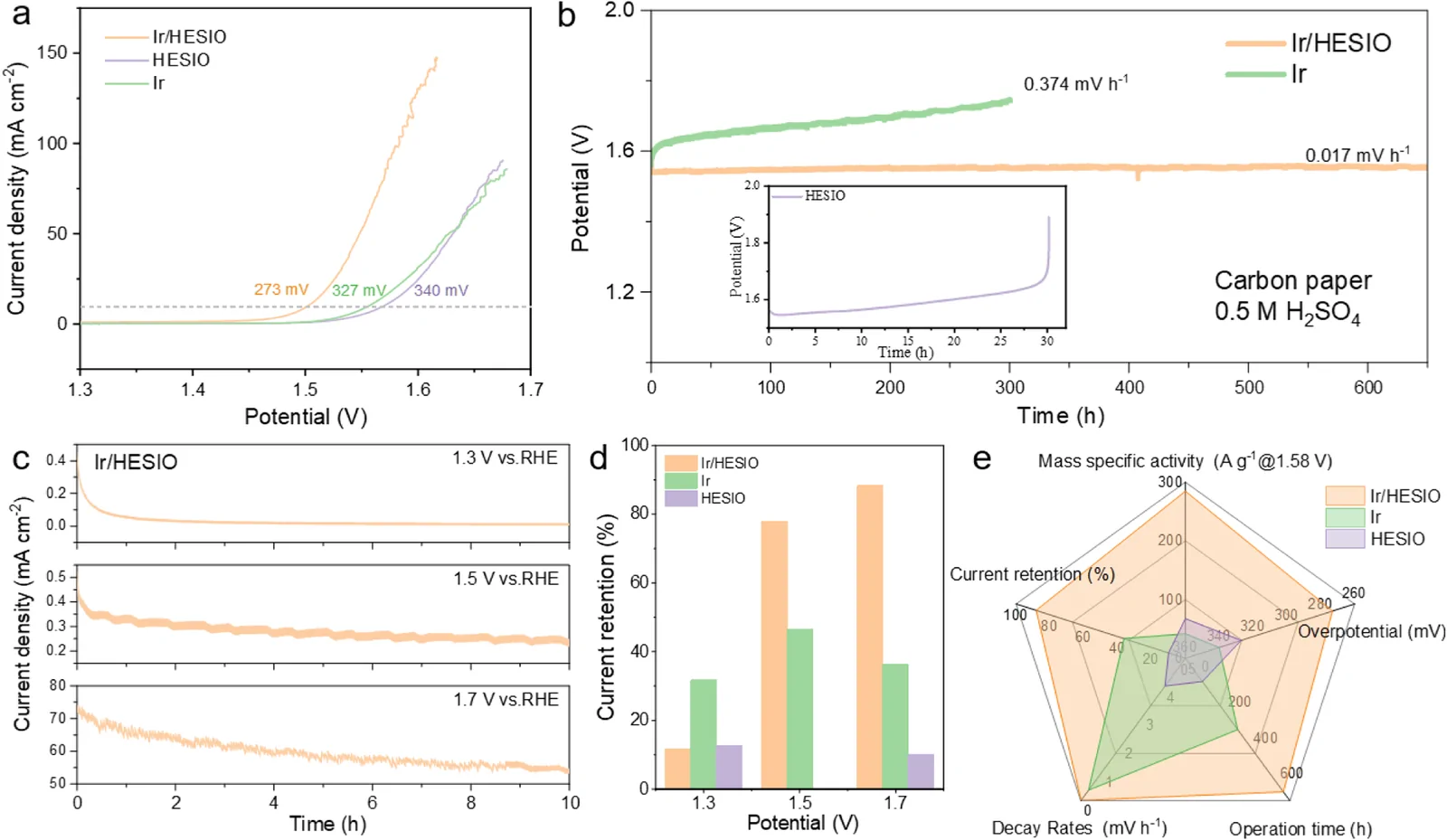
(a) LSV curves for as-prepared catalysts. (b) CP curves for as-prepared catalysts. (c) CA curves for Ir/HESIO catalysts in different constant potentials. (d) Comparison of current retention in a 2.5 h CA test for as-prepared catalysts. (e) Radar chart comparing catalytic performance across five different key indicators.

Beyond catalytic activity, operational stability is a key parameter for performance assessment. The operational durability of the catalysts was examined under both galvanostatic and potentiostatic conditions. The chronopotentiometric (CP) tests at 10 mA cm^–2^ show that the HESIO and Ir catalysts undergo a rapid potential increase within 30 and 300 h, respectively, indicating severe degradation or detachment of active sites. In contrast, Ir/HESIO maintains a stable recorded potential for over 650 h with a negligible decay rate of only 0.017 mV h^–1^ (Figure 3b). Notably, the posttest electrolyte analysis reveals that the dissolution rates of iridium species were only 0.04 μg h^–1^ for Ir/HESIO (versus 4.36 μg h^–1^ for HESIO and 0.06 μg h^–1^ for the Ir catalyst), confirming the exceptional corrosion resistance of Ir/HESIO catalyst. Besides, the high-entropy perovskite-supported Ir structure is maintained in post-CP Ir/HESIO, as evidenced by the TEM images (Figure S13), demonstrating its structural stability. The stability was further probed via chronoamperometry (CA) at various potentials ranging from 1.3 to 1.7 V vs RHE (Figure 3c, Figure S14). While the reference samples show rapid current decay under high potential, Ir/HESIO maintains a high current density throughout testing. This superiority is quantified in Figure 3d, which summarizes the current retention at different applied voltages. Ir/HESIO retains over 88% of its initial current density at a high potential of 1.7 V, whereas the Ir and HESIO control samples drop below 37% and 10%, respectively. Both CP and CA results underscore the structural robustness of the rooted-type catalysts under harsh electrochemical conditions.

For a holistic comparison, a radar chart^43^ that integrates five key performance indicators was constructed: overpotential at 10 mA cm^–2^, mass-specific activity, potential decay rate, operational duration, and current retention ratio (Figure 3e). The Ir/HESIO catalyst occupies the largest area within the radar chart, demonstrating outstanding superiority across all metrics. These results validate the multiscale stabilization strategy as an effective route to engineer highly active and durable catalysts for acidic water oxidation.

### In Situ and Theoretical Insights into Stabilization Mechanisms

To holistically probe structural evolution under operating conditions, in situ X-ray absorption spectroscopy (XAS)^44,45^ and quasi-in situ X-ray photoelectron spectroscopy (XPS) were conducted on the rooted-type Ir/HESIO and its counterparts. The XANES spectra track the average valence-state change of iridium during anodic polarization (Figure 4a). For the unsupported Ir catalyst, the white-line peak intensity at the Ir L_3_-edge gradually increases as the applied potential is raised from the open-circuit potential (OCP) to 1.7 V, indicating the continuous oxidation of the Ir element. In contrast, the peak intensity for Ir/HESIO rises slightly at 1.3 and 1.5 V and then reversibly decreases upon further increases in applied potential, even up to 1.7 V. This indicates that the presence of the perovskite substrate effectively suppresses the excessive oxidation of surface iridium nanoparticles at a high applied potential. Consistent with the valence-state trend, the corresponding in situ EXAFS spectra of the unsupported Ir catalyst show continuous enhancement of Ir–O coordination following an increase applied voltage, whereas the Ir–O coordination in Ir/HESIO remains largely unchanged (Figure 4b). Furthermore, in situ XAS spectra of both the Ti and Ni K-edges show a distinct potential-dependent elevation in the valence state of both cations (Figure S15a–b), whereas the progressive increase in Ir valence is inhibited. Notably, the valence increase for Ni is significantly more pronounced and persistent than that observed for Ti, strongly supporting the distinct roles of the cations, as revealed by the single-doping experiment. This cooperative redox behavior between Ir, Ni, and Ti clearly demonstrates that the perovskite host exerts a favorable dynamic regulatory effect on the iridium loading layer, effectively preventing adsorption saturation and overoxidation. The in situ XANES results of the HESIO catalyst indicate that the Ir oxidation state in the support still undergoes a continuous increase at an applied potential of 1.7 V. The gradual increase in the Ir–O coordination number during the OER process is also observed by in situ EXAFS at the Ir L_3_-edge for the HESIO catalyst (Figure S15c–d). This further indicates that the reversible reduction in the oxidation state of the Ir sites in the Ir/HESIO catalyst is attributed to the favorable modulation effect of the support rather than the potential change in the proportion of the two Ir species in the catalyst.

**Figure 4:**
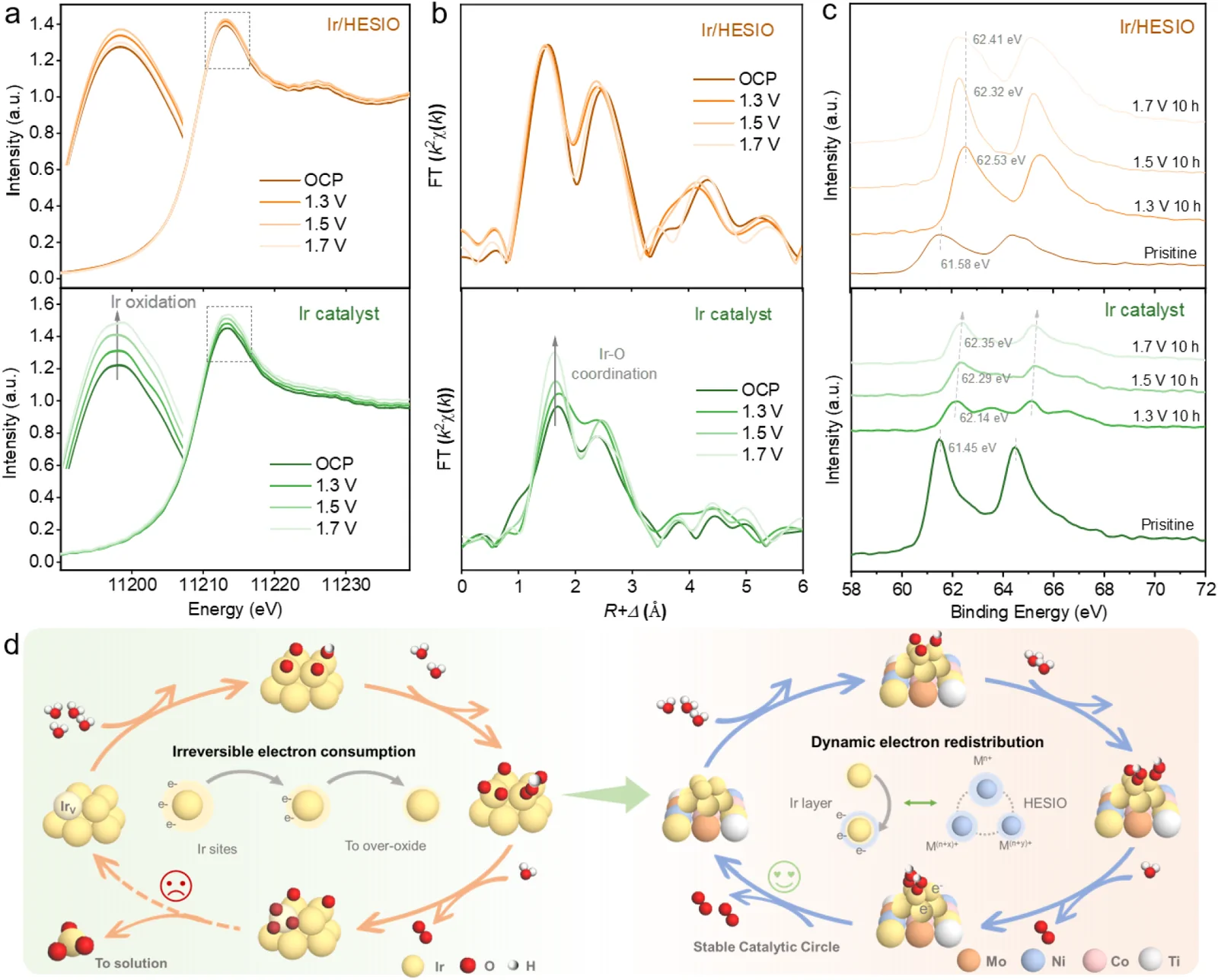
(a) The in situ XANES spectra at different applied potentials for Ir/HESIO and Ir catalysts. (b) The in situ EXAFS spectra at different applied potentials for Ir/HESIO and Ir catalysts. (c) The quasi-in situ XPS spectra at different applied potentials for Ir/HESIO and Ir catalysts. (d) The stabilization-mechanism scheme of Ir/HESIO (right) compared to the unsupported Ir cluster (left).

While XAS reflects the average coordination environment, surface-sensitive insights are more crucial for supported catalysts. Therefore, a quasi-in situ XPS experiment was further employed to monitor the near-surface electronic structure change of the Ir active layer. For the unsupported Ir catalysts, the Ir 4f binding energy shifts continuously from 61.45 eV in the pristine state to 62.35 eV at 1.7 V. For the Ir/HESIO catalyst, the binding energy increases from 61.58 eV in the pristine state to 62.53 eV at 1.3 V, then slightly decreases to 62.32 eV at 1.5 V and remains relatively stable thereafter (Figure 4c). This reversible shift at high potential provides further evidence of the in situ electronic redistribution induced by high-entropy perovskite support. More importantly, the Ir in post-650 h CP Ir/HESIO exhibits a similar valence state, with a binding energy of about 62.31 eV, to that after short-term CA treatment rather than showing excessive oxidation during the long-term operation process (Figure S16). This result indicates that the proposed dynamic electronic redistribution effect continuously works, even during long-term operation. Collectively, the above in situ spectroscopic results confirm that the support in the rooted-type catalysts effectively buffers the electronic structure of the active Ir layer, whereas the unsupported Ir catalyst undergoes progressive oxidation-driven deactivation (Figure 4d). These observations provide direct evidence of the dynamic regulatory effects during the reaction process.

To further elucidate the fundamental origin of the remarkable stability of Ir/HESIO, we investigated the reaction mechanism and performed theoretical simulations. By carrying out differential electrochemical mass spectrometry (DEMS) tests and pH-dependent tests, the intrinsic OER mechanism among different catalysts was determined. The involvement of lattice oxygen was probed in 0.5 M H_2_SO_4_ prepared with H_2_^18^O, with the ratio of ^16^O/^18^O reflecting the lattice oxygen mechanism contribution (LOM ratio).^46,47^ The Ir/HESIO and Ir catalysts exhibit low LOM ratios (about 5.4% and 10%, respectively), significantly lower than that of untreated HESIO (20%) (Figure S17). This indicates that the exsolution strategy favors a non-LOM pathway. Furthermore, the Ir/HESIO and Ir catalysts show pH-independent OER kinetics at 1.58 V compared to typical LOM kinetics in HESIO (Figure S18).^48^ Therefore, the OER pathway in Ir/HESIO and Ir catalysts can be determined to be the adsorbate evolution mechanism (AEM). Particularly, the extremely low LOM contribution in Ir/HESIO underpins its outstanding structural stability by minimizing lattice oxygen participation. The low LOM ratio in Ir/HESIO originates from the weakening of M–O covalent hybridization and lattice oxygen reactivity due to changes in the oxygen coordination environment following segregation, as indicated by the O K-edge XAS (Figure S19) and theoretical O 2p projected density of states (PDOS, Figure S20).

On the basis of the experimental results, theoretical simulations were conducted following the AEM pathway to verify the underlying catalytic mechanism. First, surface Pourbaix diagrams^49^ were constructed using our CatMath platform^50^ to identify the favorable surface state (i.e., the OER-induced surface coverage) of Ir/HESIO under operating conditions. The one-dimensional (1D) and two-dimensional (2D) surface Pourbaix diagrams (Figure 5a and b) reveal that the Ir/HESIO surface is covered by a 1-monolayer (ML) oxygen species (O*) under typical acidic OER potentials (*U* > 1.23 V). In addition, an Ir cluster supported on IrO_2_ was used as the reference model to better represent oxidized Ir species under an OER environment (Figure S21). Its surface Pourbaix diagrams also reveal a similar coverage situation of 1 ML of O* (Figure S22). The electronic states of pristine Ir/HESIO and 1 ML O*-covered Ir/HESIO were compared. The PDOS of Ir 5d orbitals (Figure 5c) reveals that O* adsorption induces a noticeable downshift of the Ir d-band center, reflecting significant electronic reconstruction of the Ir active sites under reaction conditions. On the basis of the 1 ML O*-covered Ir/HESIO and 1 ML O*-covered Ir/IrO_2_ models, an AEM-based OER pathway analysis was conducted, involving the key intermediates *OH, *O, and *OOH. The OER free-energy profiles (Figure S23) at U = 1.23 V show that the potential-determining step is the formation of *OOH from *O. For Ir/HESIO, the corresponding uphill free-energy change is 1.224 eV, which is lower than those of HESIO and Ir/IrO_2_. These results indicate that Ir/HESIO provides more favorable energetics for the adsorbate-mediated OER pathway. Furthermore, an advanced OER microkinetic volcano model,^51^ integrating potential-dependent kinetics and thermodynamic descriptors of the OER process, was derived to evaluate the catalytic performance. As shown in Figure 5d, the O*-covered Ir/HESIO surface is shifted closer to the *Sabatier* optimum compared with pristine Ir/HESIO, demonstrating optimized adsorption energetics of OER intermediates. Moreover, the incorporation of Ir clusters enables Ir/HESIO to reside in a more favorable region of the volcano model than HESIO, confirming an enhanced intrinsic OER activity that aligns with the experimental results. Most importantly, we compared the thermodynamics of progressive oxidation on the Ir/HESIO model with those on the Ir/IrO_2_ model to reveal the stabilizing mechanism. The Gibbs free-energy profiles (Figure 5e) show that the formation of highly oxidized IrO_*x*_ species is less favorable on 1 ML O*-covered Ir/HESIO than on 1 ML O*-covered Ir/IrO_2_, indicating that the HESIO support can suppress excessive oxidation of Ir under OER-relevant conditions. To further explain the origin of this difference, we conducted charge-density-difference and Bader charge analyses (Figure S24). The Ir/HESIO model exhibits a larger interfacial charge transfer than that of Ir/IrO_2_, revealing stronger electronic coupling between the Ir cluster and the HESIO support. This interfacial charge redistribution can partially compensate for electron depletion around Ir during oxidation, thereby reducing the tendency toward overoxidation.

**Figure 5:**
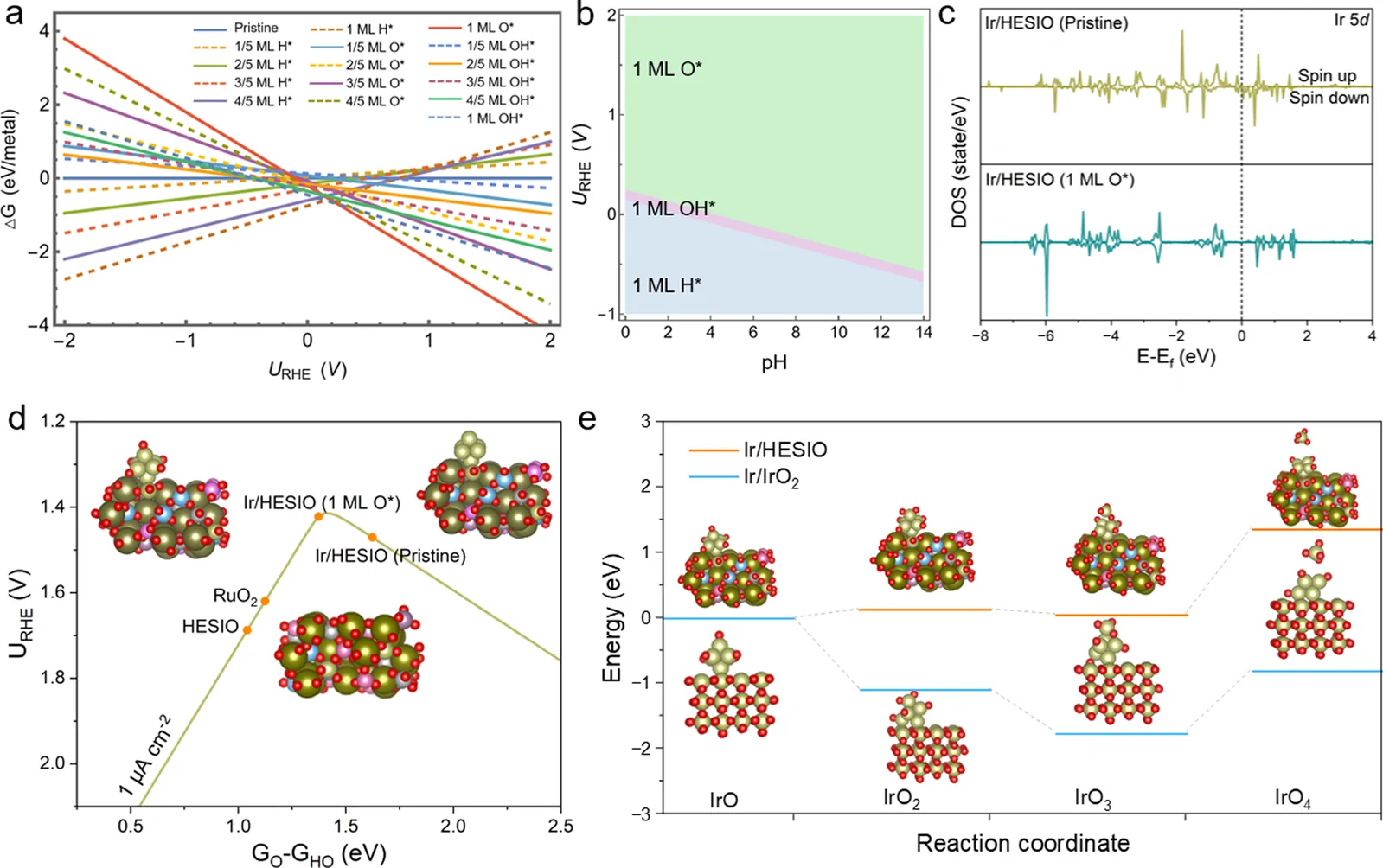
(a) Calculated one-dimensional (1D) surface Pourbaix diagram as a function of potential vs RHE (reversible hydrogen electrode) at 298.15 K. (b) Corresponding two-dimensional (2D) surface Pourbaix diagram as a function of potential vs RHE (reversible hydrogen electrode) and pH at 298.15 K for the Ir/HESIO model. (c) Projected density of states (PDOS) of the Ir 5d orbitals for the active Ir site on pristine Ir/HESIO and Ir/HESIO with 1 ML O* coverage. The Fermi level is set to zero. (d) Microkinetic OER volcano model derived at a current density of 1 μA cm^–2^, showing the predicted catalytic activity of different catalysts. Insets illustrate the surface models used in the calculations. (e) The Gibbs free energy changes associated with progressive overoxidation for the Ir/IrO_2_ and the Ir/HESIO models.

Considering the consistency between the basic characterization, in situ spectroscopy results, and theoretical simulation, we confirm that the excellent stability of Ir/HESIO intrinsically originates from the high-entropy effect in the following three aspects: (i) the sluggish diffusion characteristic governs the exsolution process to form a rooted-type structure, where the active loading layer is effectively anchored in the HESIO support; (ii) this rooted architecture provides the foundation to promote the geometric confinement of the active sites and the intimate interfacial contact channel; (iii) the inherent electronic flexibility of the high-entropy lattice allows it to function as an electronic buffer layer to dynamically regulate the electronic state of the active sites under operating conditions, inhibiting excessive adsorption and overoxidation.

### Applicability Evaluation of Ir/HESIO in a Proton-Exchange-Membrane Electrolyzer

The practical applicability of the Ir/HESIO catalyst was assessed in a proton-exchange-membrane electrolyzer system. Ir/HESIO serves as the anode of a membrane electrode assembly (MEA) with a commercial Pt/C catalyst used for the cathode. The cross-sectional SEM image of the MEA reveals a well-defined and uniform catalytic layer on both the anode and cathode sides (Figure 6a). The corresponding EDS mapping (Figure S25) further confirms the homogeneous distribution of iridium throughout the anode catalytic layer, which is crucial for the efficient utilization of the active sites. Polarization curves (Figure 6b) recorded with varying Ir loadings demonstrate the outstanding activity of Ir/HESIO in MEA systems. To be specific, it requires low cell voltages of only 1.71 and 1.80 V to achieve current densities of 2 A cm^–2^ and 3 A cm^–2^, respectively, with just 0.525 mg_Ir_ cm^–2^. Ir/HESIO also exhibits excellent activity with an ultralow Ir loading of 0.284 mg_Ir_ cm^–2^, where the single cell just requires 1.73 V to achieve 2 A cm^–2^. To deconvolute voltage losses, the polarization curve was analyzed employing the high-frequency resistance (HFR) measurements (Figure 6c), separating the total overpotential into ohmic, kinetic, and mass-transport contributions. At 1 A cm^–2^ and 2 A cm^–2^, the kinetic overpotentials for Ir/HESIO are merely 321 mV and 337 mV, respectively, highlighting its favorable reaction kinetics (Figure S26).

**Figure 6:**
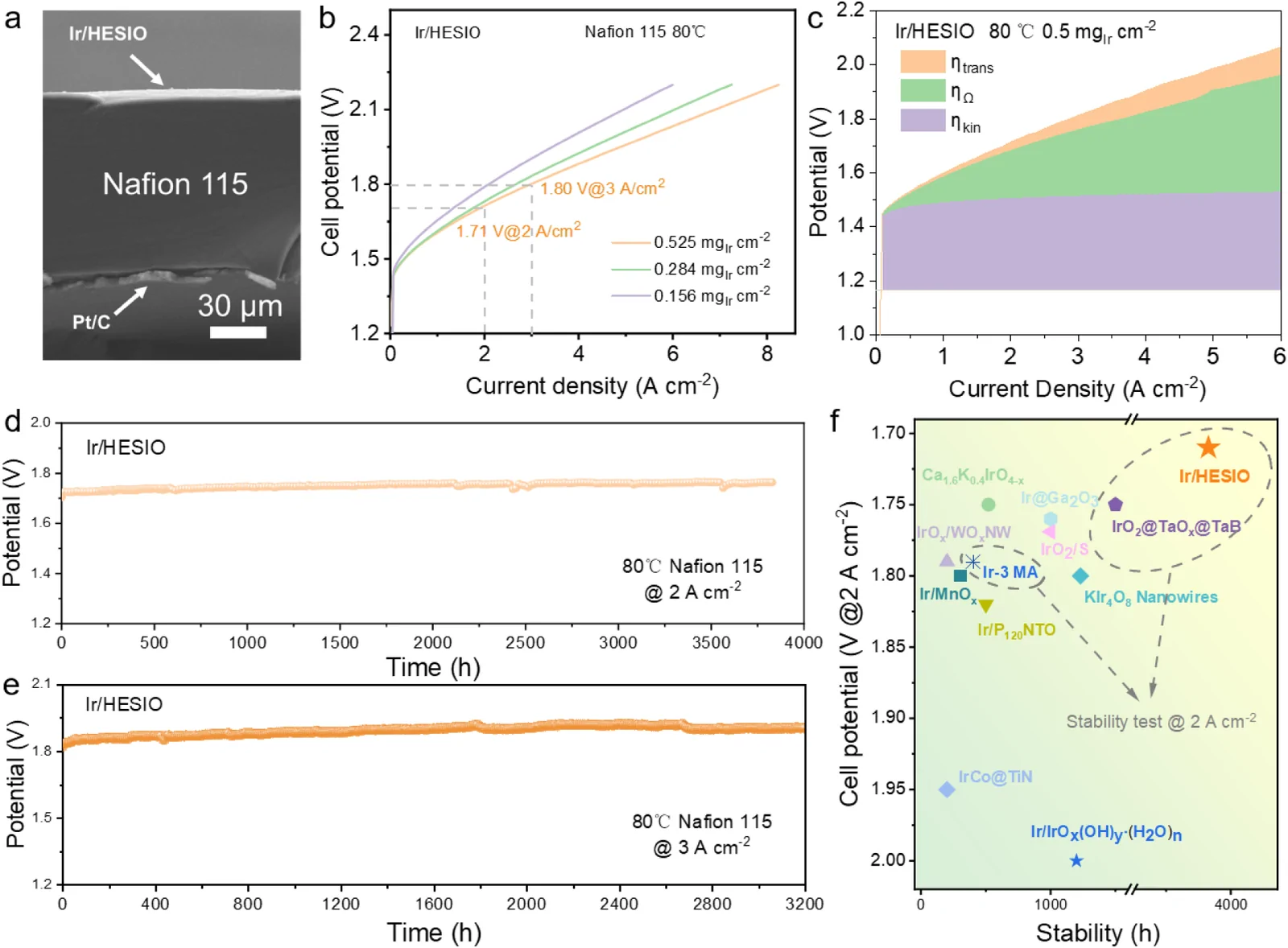
(a) The cross-sectional SEM image of the MEA fabricated by Ir/HESIO. (b) The polarization curves of MEAs with different anode Ir loadings. (c) The deconvoluted polarization curve according to the HFR measurements. (d) The stability test at a constant current density of 2 A cm^–2^. (e) The stability test at a constant current density of 3 A cm^–2^. (f) The activity and stability of Ir/HESIO compared with earlier reports.

Durability, a decisive parameter for practical water electrolysis, was systematically assessed through prolonged galvanostatic tests. Ir/HESIO exhibits exceptional durability in the MEA configuration, operating steadily at a demanding current density of 2 A cm^–2^ for over 3800 h. During the period from 500 to 3800 h, the average voltage decay rate is only 7.19 μV h^–1^ (Figure 6d). The stability was further evaluated under more aggressive conditions at 3 A cm^–2^, where the Ir/HESIO-based electrolyzer maintains stable operation for over 3200 h with an overall potential decay lower than 4.7% (Figure 6e). To probe the intrinsic stability of Ir/HESIO, posttest analysis was conducted. Deconvolution of the polarization curves after 3800 h of operation reveals a minimal decrease in the kinetic overpotential (Figure S27), and comparison of the overpotential contribution ratios before and after testing confirms that kinetic losses remain largely unchanged (Figure S28, Table S3). This indicates that the intrinsic activity of Ir/HESIO is well preserved during long-term operation, effectively resisting degradation under harsh acidic and oxidizing environments. The reason for the change in overpotential contributions is further revealed by the morphology of the cross-sectional MEA. The compressed catalytic layer distorts the pore networks, increasing the mass transport overpotential after long-term testing. However, the SEM image also reveals enhanced catalyst layer-proton exchange membrane interface contact (yellow line in Figure S29) and the microwrinkling of the catalytic layer, optimizing the accessibility of active sites and thus improving catalytic kinetics during water splitting. The outstanding durability originates from the unique “rooted-type” structure formed via phase segregation and the dynamic electron regulation provided by the HESIO support. Consequently, the Ir/HESIO resides in the top-right region in the activity–stability Ashby plot (Figure 6f, Table S4), outperforming state-of-the-art Ir-based OER catalysts in both activity and exceptional long-term stability,^52–62^ thereby positioning it as a highly promising anode for industrial PEM water electrolysis. Besides, the performance comparison between Ir/HESIO and the widely recognized rutile IrO_2_ benchmark further demonstrates the practical applicability of this catalyst (Figure S30). Furthermore, it must be acknowledged that the catalysts reported earlier have demonstrated extremely outstanding performance,^63,64^ but the Ir/HESIO catalyst still exhibits robust stability under high current-density conditions. At the same time, this work has provided an innovative multiscale stabilization mechanism that combines the catalyst’s bulk stability with its dynamic adaptability under reaction conditions.

## Conclusion

In summary, we provided an innovative rooted-type catalyst supported by high-entropy perovskite via post-treatment segregation based on the perovskite bulk. According to experimental characterization, the rooted structure has been confirmed, featuring uniformly dispersed and embedded active iridium clusters in the support substrate, thereby strongly anchoring the supported nanoparticles. In situ spectroscopy experiments and theoretical calculations reveal the stabilization mechanism of rooted-type catalysts under operating conditions. The high-entropy perovskite could dynamically regulate the electronic environment surrounding the supported iridium active layer, thus suppressing the excessive adsorption and deep oxidation of active sites. Therefore, the Ir/HESIO catalysts were stabilized by the multiscale mechanism of a physically strong anchor and in situ dynamic modulation. The PEMWE single cell fabricated by Ir/HESIO continuously operates for over 3800 h at a current density of 2 A cm^–2^ with just 2.2% potential decay, and also stably operates for over 3200 h at a higher current density of 3 A cm^–2^ with just 4.7% potential decay. This work reports a novel rooted-type catalyst with practical activity and stability and establishes a dynamic stabilization strategy, offering a versatile platform for heterogeneous catalysts toward OER and beyond.

## Supplementary Material



## Notes

Additional experimental details, materials and methods; comprehensive characterization and performance evaluation figures; and supplementary comparison tables (PDF)
