# Supplementary material for: Dynamic Electron Redistribution on Rooted-Type High-Entropy Perovskite-Supported Ir Catalysts for Durable Proton-Exchange-Membrane Water Electrolysis
Source: J Am Chem Soc. 2026 Aug 29;148(36):39187–98. doi: 10.1021/jacs.6c13780 (PMC13587660; doi:10.1021/jacs.6c13780)
Supplement: Supplementary file 1 [file ja6c13780_si_001.pdf]

Supporting Information

Dynamic Electron Redistribution on Rooted-Type High-Entropy Perovskite-Supported Ir Catalysts for Durable Proton-Exchange-Membrane Water Electrolysis

Jing Ni[1,2,#], Yuan Wang[3,#], Zhaoping Shi[1,2], Cheng Huang[1,2], Mingrui Yu[1,2], Heng Liu[3], Meiling Xiao[1,2,4,], Changpeng Liu[1,2,4,*], Hao Li[3,*], Wei Xing[1,2,4,*]*

1 Hydrogen Energy Industry Institute of Jilin Province, Changchun Institute of Applied Chemistry, Chinese Academy of Sciences, Changchun, 130022, China.

2 School of Applied Chemistry and Engineering, University of Science and Technology of China, Hefei, 230026, China.

3 Advanced Institute for Materials Research (WPI-AIMR), Tohoku University, Sendai 980-8577, Japan.

4 CIAC - HKUST Joint Laboratory for Hydrogen Energy, Changchun, 130022, China.

*Corresponding author.

E-mail: mlxiao@ciac.ac.cn (Xiao M.); liuchp@ciac.ac.cn (Liu C.); li.hao.b8@tohoku.ac.jp (Li H.); xingwei@ciac.ac.cn (Xing W.)

[#] These authors contributed equally to this work.

Contents:

Experimental Section

Supplementary Figures and Tables: Figure S1 to S30, Table S1 to S4

References

1. Experimental Section

Chemicals and reagents

All chemicals and reagents were obtained from commercial suppliers and were not further purified before use. Titanium isopropoxide (TTIP) and cobalt chloride hexahydrate ($CoCl_2 \cdot 6H_2O$) were purchased from Shanghai Macklin Biochemical Technology Co., Ltd. Dipotassium hexachloroiridate (K_2IrCl_6) was obtained from Shanghai Aladdin Biochemical Technology Co., Ltd. Hexaammonium heptamolybdate tetrahydrate ($(NH_4)_2Mo_7O_{24} \cdot 4H_2O$) tetrahydrate, nickel chloride hexahydrate ($NiCl_2 \cdot 6H_2O$) and ethylene glycol (EG)were purchased from Xilong Chemical Co., Ltd. Strontium nitrate ($Sr(NO_3)_2$) and citric acid ($C_6H_8O_7$) were obtained from Beijing Chemical Factory. Commercial Pt/C (40 wt% Pt) catalyst was obtained from Johnson Matthey Company. 5 wt% Nafion® ionomer and Nafion 115 membrane were purchased from DuPont Co.

Preparation of HESIO high-entropy perovskite

The HESIO high-entropy perovskite was prepared by a sel-gol method referenced from earlier reports[1-2]. Specifically, 280 mg $C_6H_8O_7$, 480 mg $Sr(NO_3)_2$ and 55 mg K_2IrCl_6 were dissolved in 10 mL of deionized water. Further, $NiCl_2$, $CoCl_2$ and $(NH_4)_2Mo_7O_{24}$ with an equal molar ratio to Ir were added to the above solution while stirring to form solution A. 32 μL TTIP was mixed with 4 mL EG to form solution B. Then, solution A was added dropwise to solution B under stirring to form solution C. After being stirred at 70℃ for 3 hours and then dried at 180°C in an oven for 12 hours, the sol precursor was obtained. Finally, the above-mentioned sol was calcined to obtain a high-entropy perovskite catalyst. The specific calcination procedure was as follows: it was successively calcined at 200°C and 300°C for 6 hours each, then at 500°C for 3 hours, and at 600°C for 6 hours. The heating rate was 5°C per minute.

Preparation of Ir/HESIO and Ir catalysts

The Ir/HESIO was obtained via the separation treatment of HESIO. Specifically, the prepared HESIO was heat-treated in a flow H_2/Ar atmosphere at 600°C for 3 hours. Then, the Ir/HESIO were obtained after washing with a 1 M HCl solution and deionized water five times, and drying at 55℃

for 12 hours.

The Ir catalysts were obtained via a similar sol-gel and separation process, but without the doped elements except for iridium.

The compared single-element doping Ir/SrIr(M)O_3 samples (M = Ni, Mo, Co, Ti) were prepared with the same process as Ir/HESIO, but changing the doped elements kinds and controlling the Ir and M with an equal molar ratio.

Structure characterization and spectroscopy test

Transmission electron microscopy (TEM) images were obtained on a JEOL JEM-2100F transmission electron microscope. The EDS line scan results were obtained using an Oxford Instruments X-Max 80T. High-annular dark-field scanning transmission electron microscopy (HAADF-STEM) and atomic resolution EDS-mapping were performed on Titan 80-300 scanning/transmission electron microscope. X-ray diffraction (XRD) patterns were collected using a D8 ADVANCE diffractometer with Cu Kα radiation in the range of 5°-90°. The ratio of different metal elements was determined by inductively coupled plasma optical emission spectroscopy (ICP-OES, Thermo Scientific iCAP 7000 SERIES). The quasi in situ surface oxidation of catalysts under OER conditions was determined by X-ray photoelectron spectroscopy (XPS) in a Thermo Fisher Scientific ESCALAB 250Xi unit with Al-Kα (hν = 1486.6 eV) as the X-ray source. The Ir L3-edge X-ray absorption spectroscopy (XAS) measurements were performed at BL14W1 and BL11B beamlines in Shanghai Synchrotron Radiation Facility (SSRF). In-situ differential electrochemical mass spectrometry (DEMS) tests were conducted on a differential electrochemical mass spectrometer purchased from Linglu Instruments (Shanghai) Co., Ltd. The test was performed in a standard three-electrode system with 0.5 M H_2SO_4 in $H_2{}^{18}O$ as electrolyte. The signal $^{34}O_2/^{36}O_2$ is defined as the ratio of the integral area of the intensity curve and is used to calculate the LOM ratio. The cross-sectional scanning electron microscopy (SEM) images were obtained on a Zeiss Sigma 300 field emission scanning electron microscope.

Electrochemical measurement in a three-electrode cell

Electrochemical performance tests of catalysts were carried out in a standard three-electrode system on CHI 760E electrochemical workstation. Typically, a Glassy carbon electrode with a diameter of 3mm coated with samples was used as the working electrode. A carbon rod was used as the counter electrode, and a Hg/Hg_2SO_4 electrode filled with saturated K_2SO_4 solution was used as the reference electrode. All of the measured potentials in this study are given referenced with a reversible hydrogen electrode (RHE) converted according to the following equation:

$$E(\text{vs. RHE}) = E(\text{vs. Hg/Hg}_2\text{SO}_4) + 0.701\ \text{V}$$

Where 0.701 V is the potential difference between the Hg/Hg_2SO_4 reference electrode and RHE in 0.5 M H_2SO_4, which was calibrated by the open circuit potential (OCP) test.

The catalysts (5 mg) were dispersed in a mixture solution of 0.45 mL ethanol and 50 μL Nafion (5 wt.%). After ultrasonication, 3.6 μL homogeneous ink was dropped on a carbon glassy electrode. Linear sweep voltammetry (LSV) was tested with a scan rate of 5 mV s^{-1} with iR correction. Chronopotentionmetry (CP) tests were conducted on carbon paper at 10 mA cm^{-2}. Chronoamperometry tests were conducted on carbon paper at a range of representative potentials (1.3-1.7 V). The pH-dependent test was performed to collect the LSV curves in the H_2SO_4 solution with different hydrogen ion concentrations.

In-situ X-ray absorption fine structure (XAFS) spectroscopy test

X-ray absorption fine structure (XAFS) spectroscopy was performed using the Super XAFS-H3000 (National Innovation Scientific Instruments (Suzhou) Co., Ltd.) in transmission mode at 30 kV and 30 mA. XAFS spectra were acquired through the transmission mode. A custom-made cell was utilized to conduct the electrochemical in situ XAFS tests. The sample is uniformly dispersed on carbon paper and immersed in a 0.5 M H_2SO_4 electrolyte. To track the evolution of the active site during the OER reaction, a range of representative potentials (1.3–1.7 V) was applied to the electrode. This comprehensive approach provides valuable insights into the structural changes of the Ir L_3-edge during electrochemical reactions.

DFT calculation

Spin-polarized density functional theory (DFT) calculations were carried out using the Vienna *Ab initio* Simulation Package (VASP)[3] with the projector augmented-wave (PAW) method[4]. The exchange-correlation interactions were treated via the generalized gradient approximation (GGA)[5] with the revised Perdew–Burke–Ernzerhof (RPBE) functional[6]. A plane-wave cutoff energy of 400 eV was employed. The Brillouin zone sampling was performed using a 2 × 2 × 1 Monkhorst–Pack k-point mesh[7]. To better describe the localized 3d electrons of Ni and Co, a Hubbard U correction was applied within the DFT+U framework[8]. The surface was modeled using a periodic slab separated by a 20 Å vacuum layer along the z-direction. During structural optimization, the bottom two layers were fixed at their bulk positions, while the upper layers were fully relaxed. The force convergence and energy convergence criteria were set as 0.05 eV $Å^{-1}$ and 10^{-5} eV $cell^{-1}$. Surface Pourbaix diagrams were constructed based on the computational hydrogen electrode (CHE) model[9] as a function of pH and potential (U). Zero-point energy (ZPE) and entropic corrections were included at 298.15 K. Furthermore, explicit solvation effects were considered for the HO* intermediate to account for hydrogen-bonding stabilization[10-11].

Applicability evaluation for the catalyst in a proton exchange membrane electrolyzer

Ir/HESIO was used as the anode catalyst and 40 wt% commercial Pt/C was used as the cathode catalyst to fabricate the membrane electrode assembly (MEA) on the two sides of Nafion 115 membrane. The catalyst was dispersed in a mixture of isopropanol, deionized water, and 5 wt% Nafion® solution with a certain ratio to obtain the ink. The catalyst ink was first sprayed on the polytetrafluoroethylene (PTFE) sheets and then pressed and transferred to the Nafion membrane. Finally, the obtained MEA was preserved in deionized water for further measurements.

Ti felt was used as a porous transport layer (PTL) to construct a PEMWE single cell, but the anode PTL was coated with a Pt layer. The cell was assembled with a pressure of 3.8 N m. The active area of the catalytic layer was about 4 cm^2. Then, the flowing deionized water was supplied to the cell as reactants. The polarization curves were collected at 80 °C. The stability of the PEMWE single cell was evaluated by CP tests at a constant current density of 2 A cm^{-2} and 3 A cm^{-2}.

2. Supplementary Figures and Tables

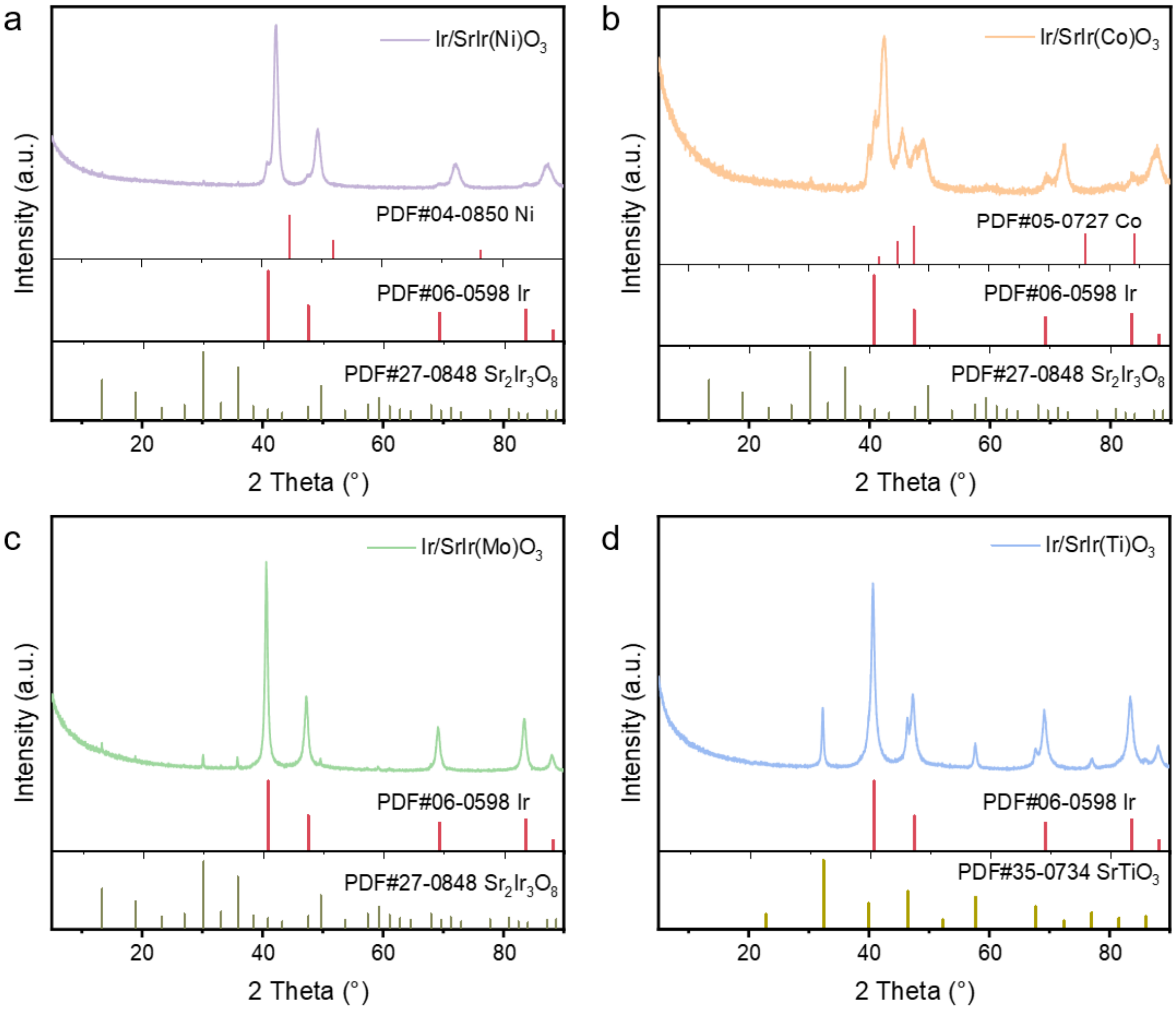

Figure S1 The XRD patterns of **a,** $Ir/SrIr(Ni)O_3$, **b,** $Ir/SrIr(Co)O_3$, **c,** $Ir/SrIr(Mo)O_3$, and **d,** $Ir/SrIr(Ti)O_3$

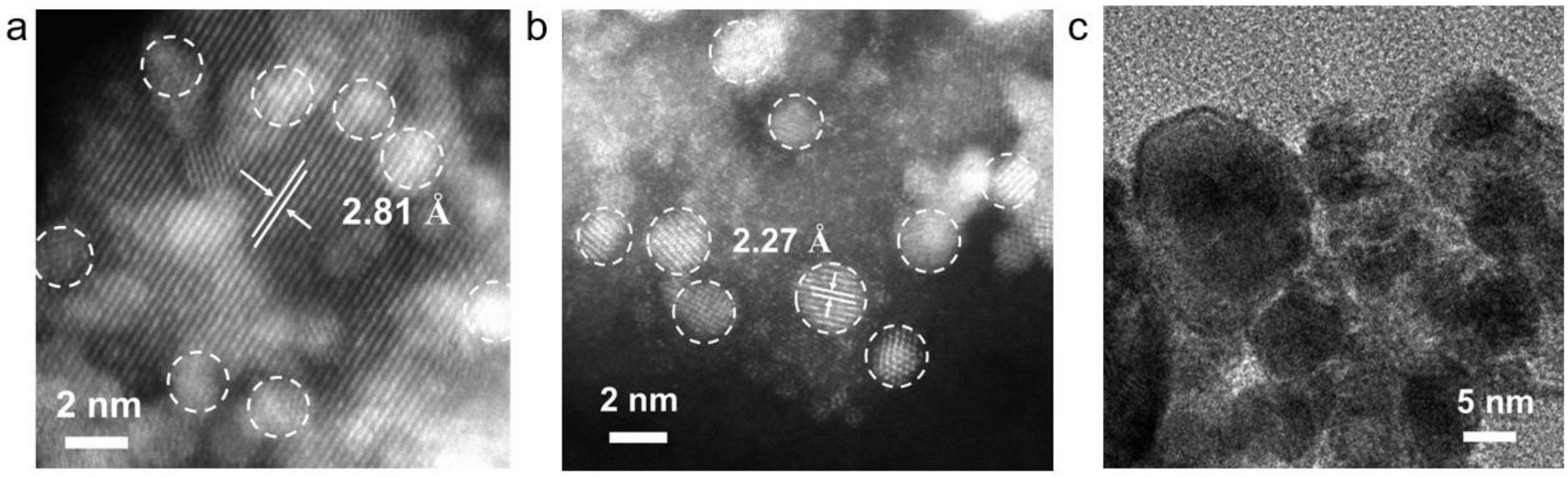

Figure S2 a, The HAADF-STEM image of Ir/HESIO, where the lattice spacing of the perovskite support was displayed, **b,** The HAADF-STEM image of Ir/HESIO, where the lattice spacing of the Ir nanoparticles was displayed, **c,** The TEM image of Ir/HESIO

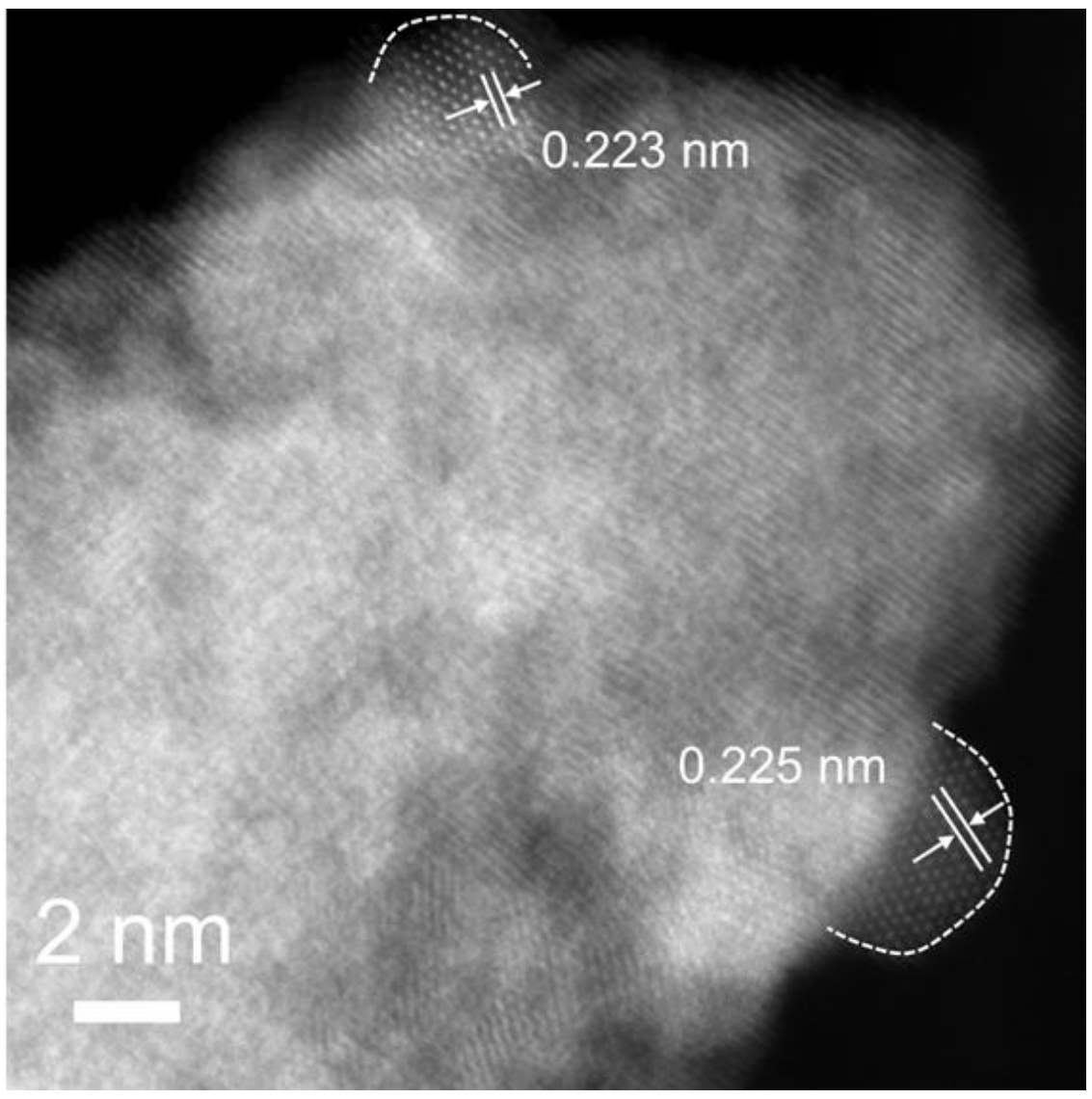

Figure S3 The HAADF-STEM image of the interface between Ir nanoparticle and the HESIO support in Ir/HESIO

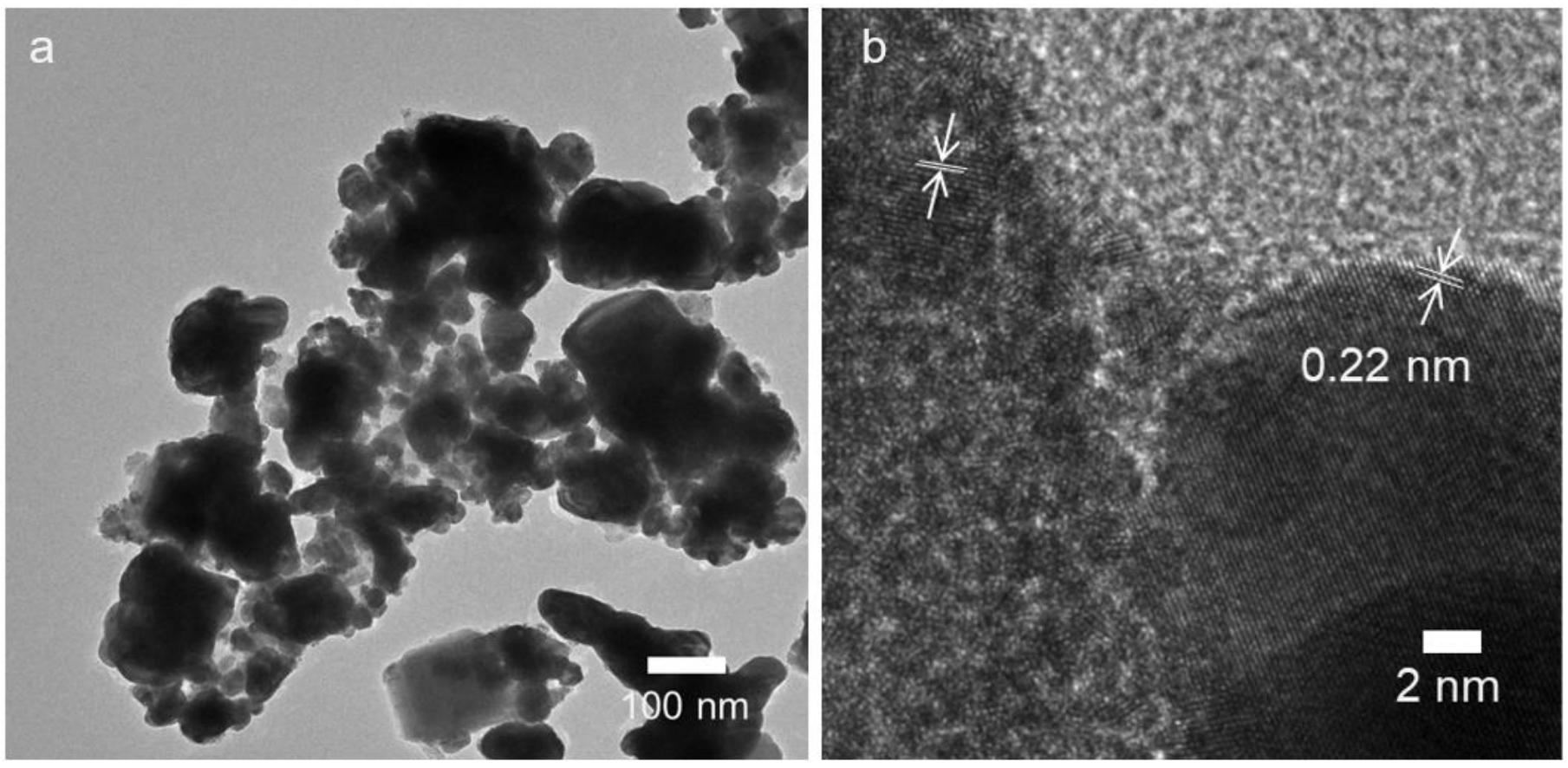

Figure S4 a, The TEM image and **b,** HRTEM image of the unsupported Ir sample

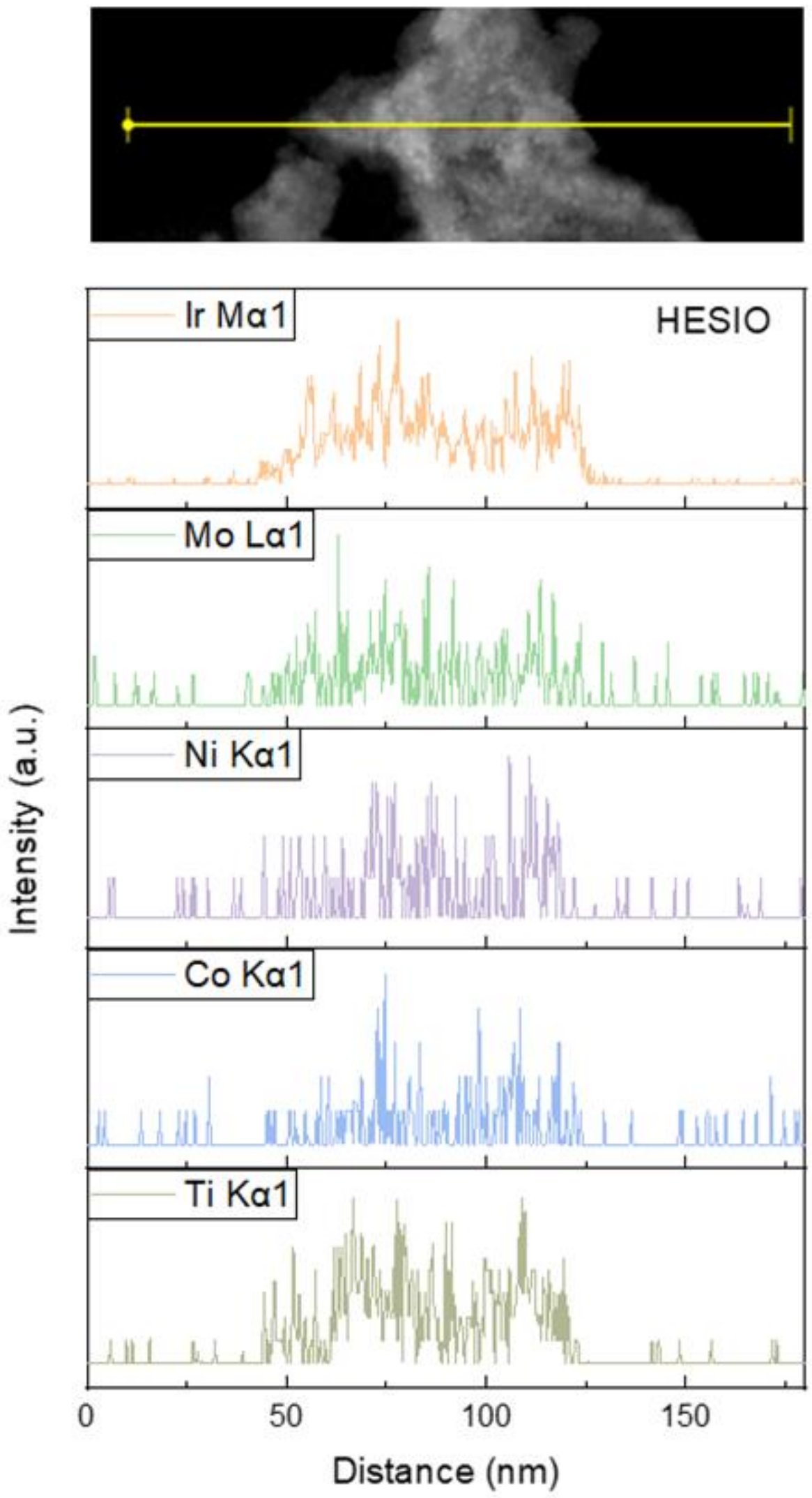

Figure S5 The EDS line-scan profile of the HESIO precursor before separation

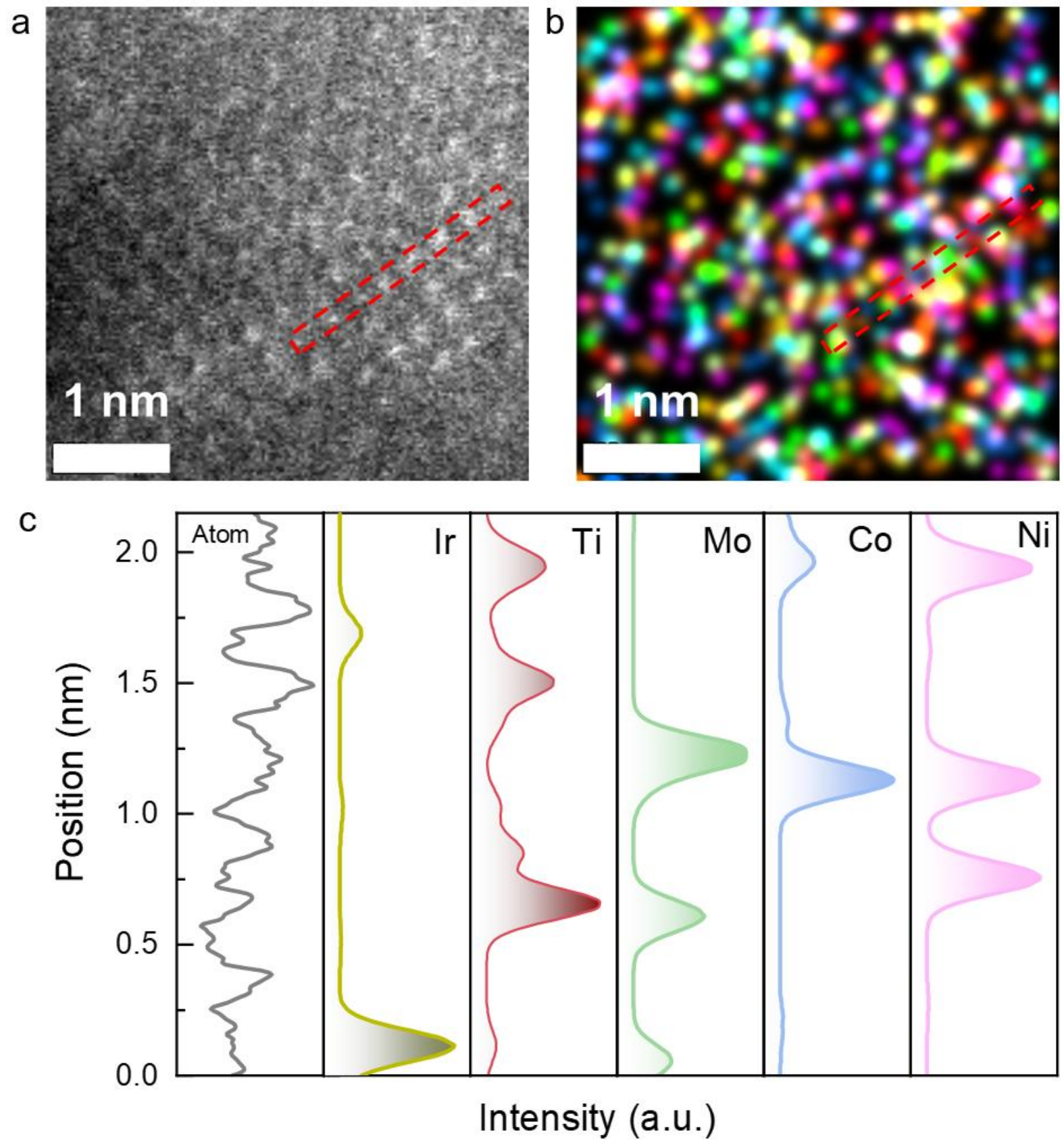

Figure S6 The EDS mapping and atomic-resolution element profile of the support region in Ir/HESIO

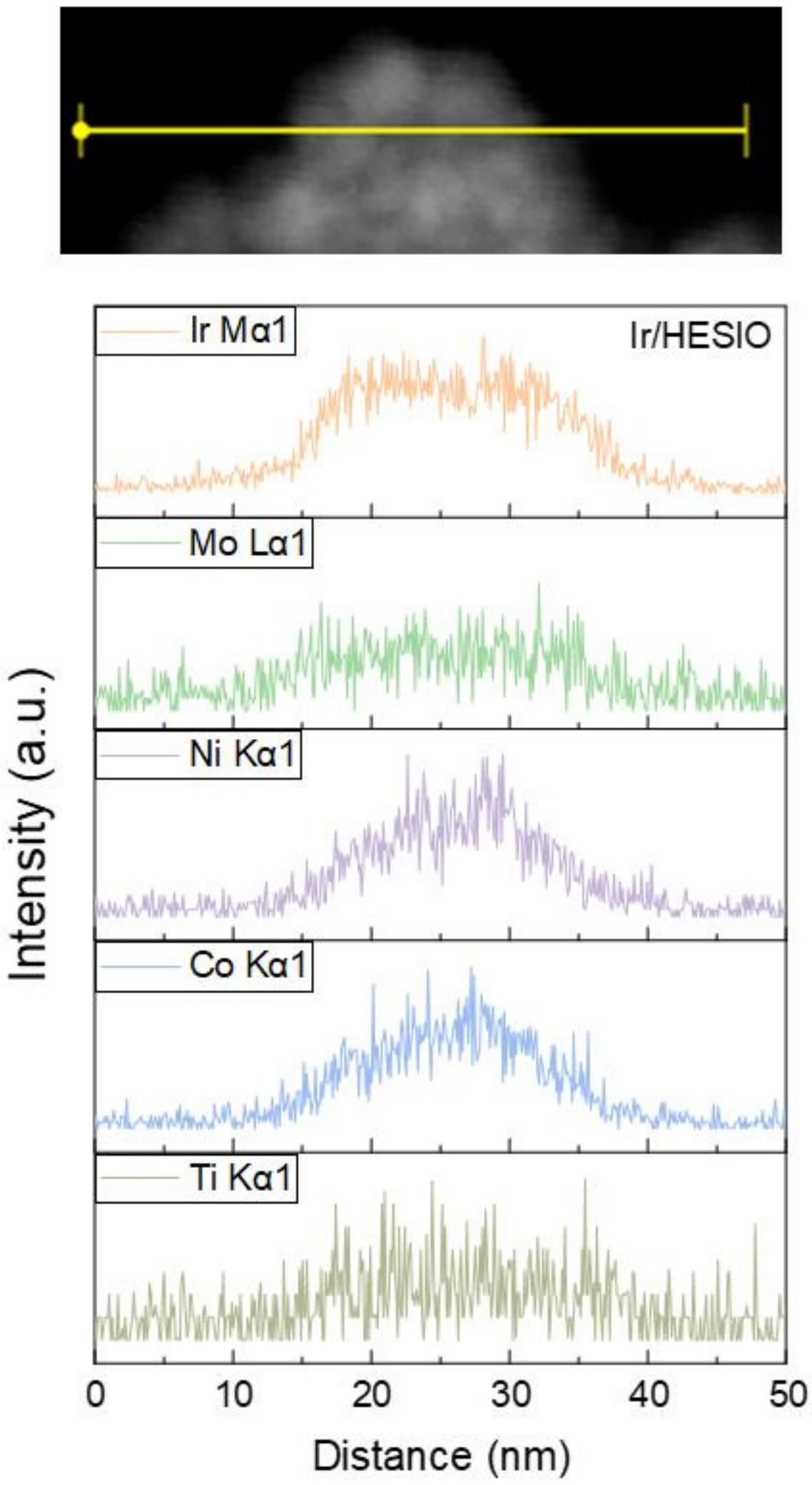

Figure S7 The EDS line-scan profile of the Ir/HESIO

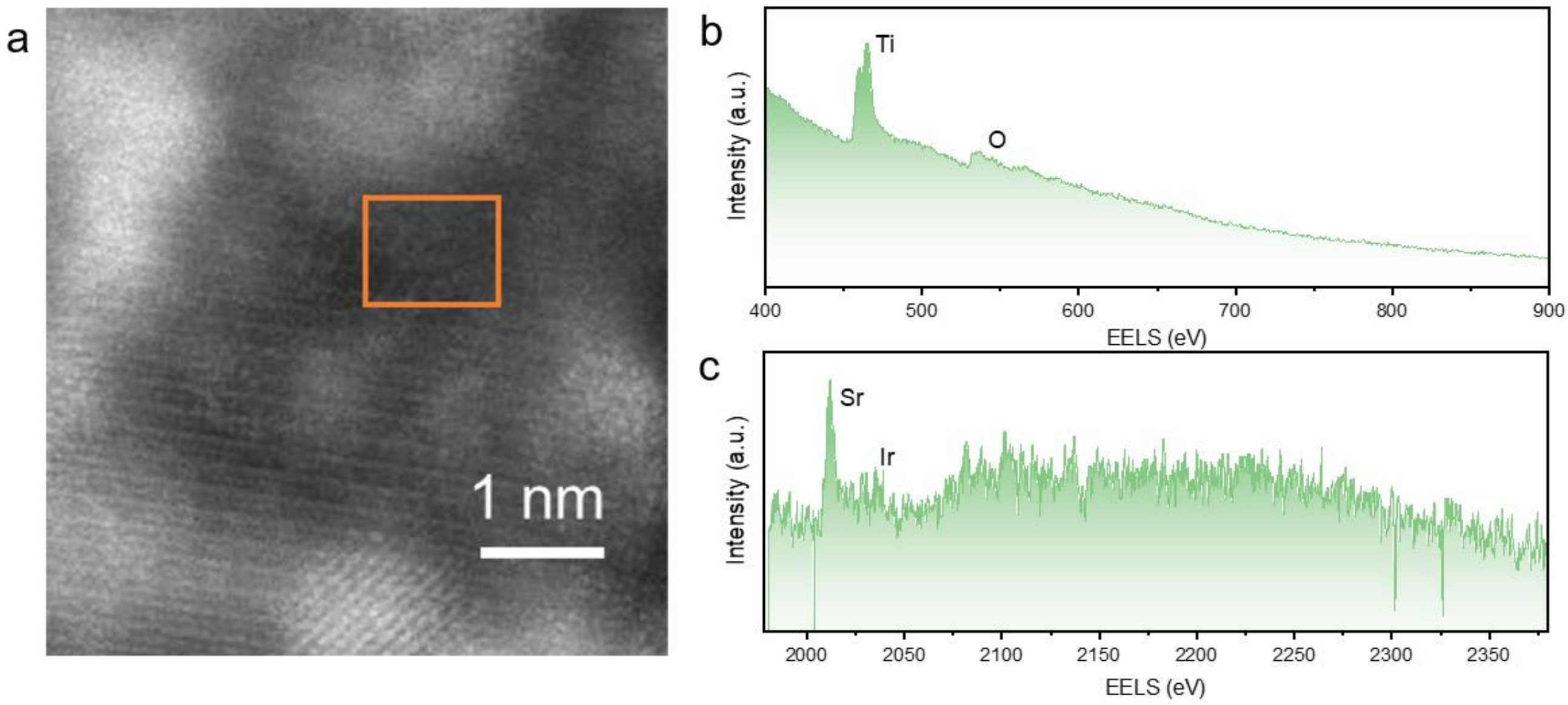

Figure S8 a, the HAADF-STEM image of Ir/HESIO, **b,c,** the EELS spectra of the yellow box area in Figure **a**

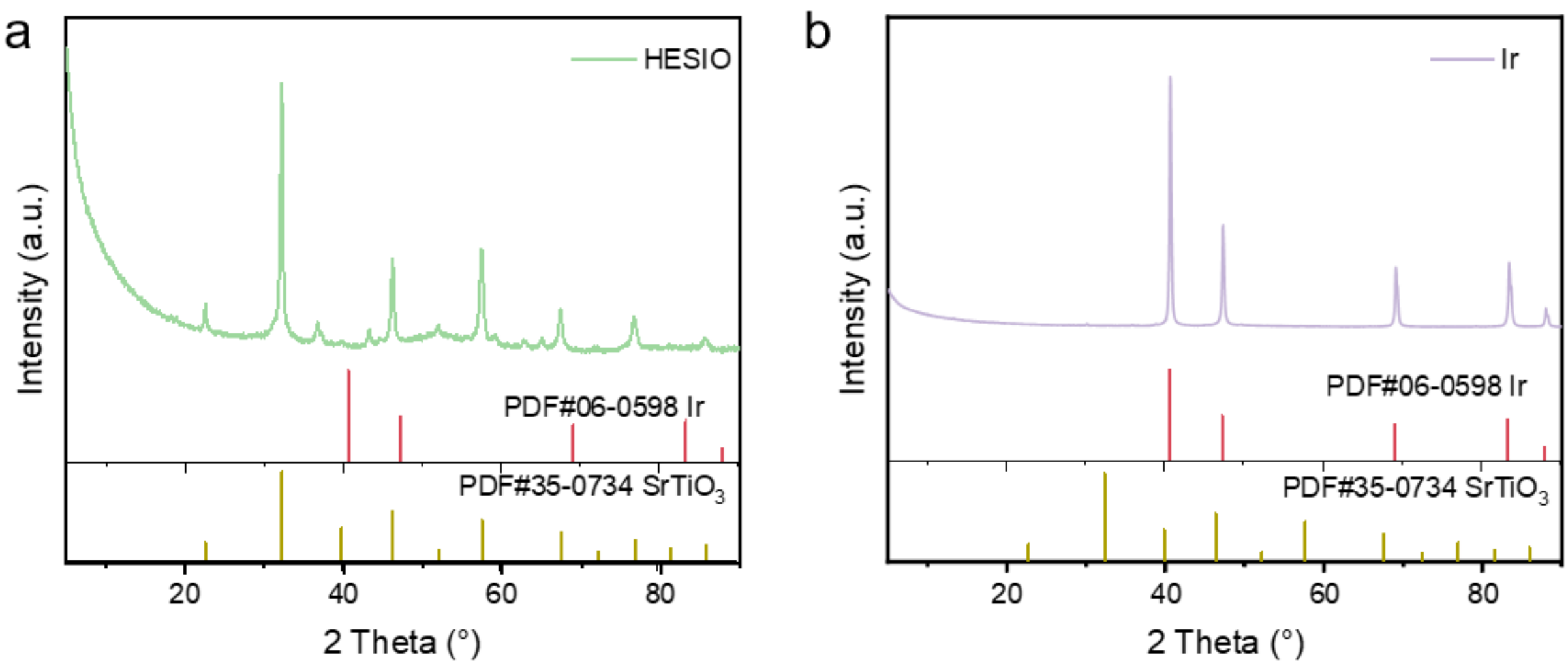

Figure S9 The XRD patterns of **a,** the untreated HESIO and **b,** the unsupported Ir catalysts

Table S1 The atomic ratio of different elements in the as-prepared catalyst

**Catalysts**	**Atom(Co)**	**Atom(Ir)**	**Atom(Ti)**	**Atom(Mo)**	**Atom(Ni)**	**Atom(Sr)**	**Ir wt.**
**HESIO**	26.10	28.17	22.66	4.53	18.54	39.79	0.41
**Ir/HESIO**	20.16	47.63	14.42	2.85	14.93	10.81	0.68

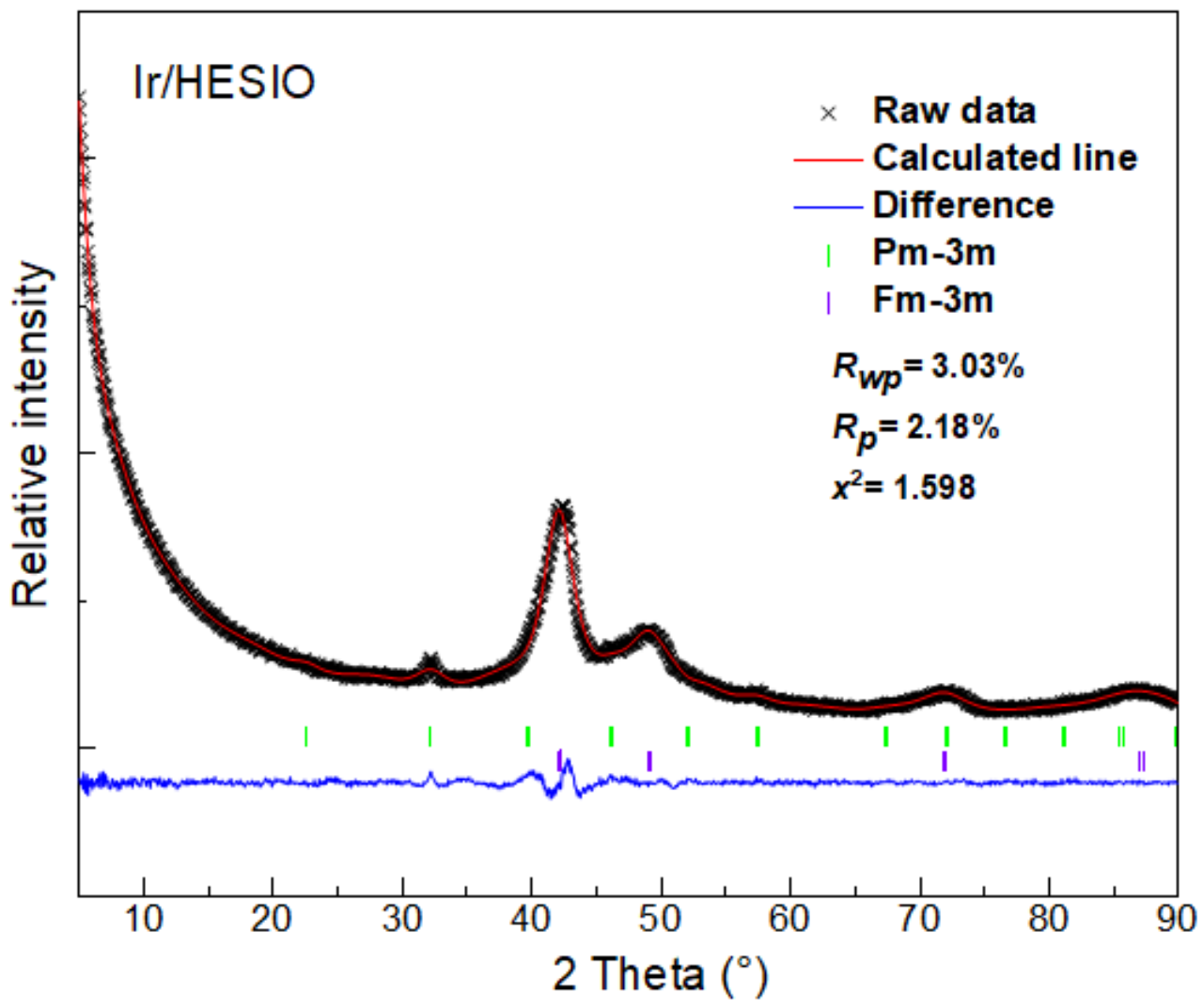

Figure S10 The XRD refinement pattern of Ir/HESIO

Table S2 The atom ratio of B-site elements in HESIO support and mass fraction of Ir element in the catalytic layer as determined by XRD refinement pattern

Catalysts	Atom(Co)	Atom(Ir)	Atom(Ti)	Atom(Mo)	Atom(Ni)	Atom(Sr)	Catalytic layer Ir wt.
**Ir/HESIO**	29.45	23.53	21.06	4.16	21.81	15.78	0.45

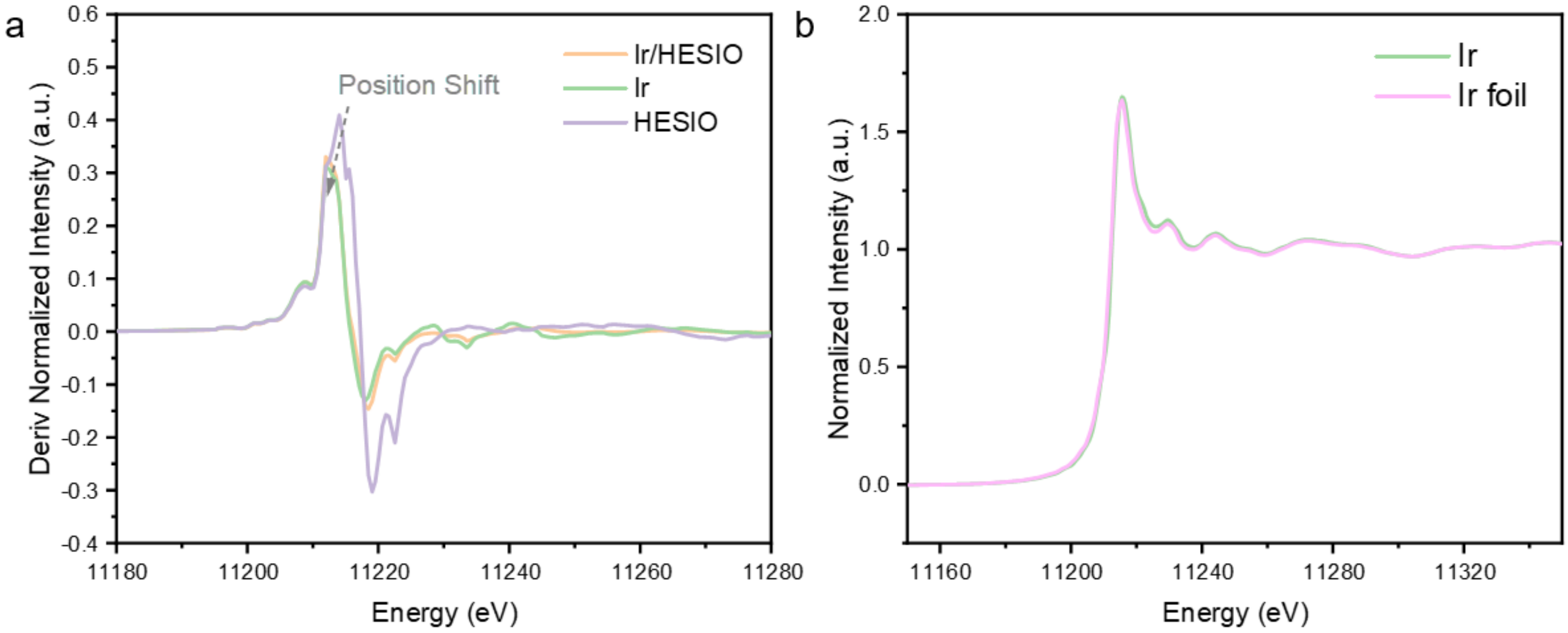

Figure S11 a, The first derivative of the XANES spectra of as-prepared catalysts; **b,** The XANES spectra for Ir catalysts and Ir foil

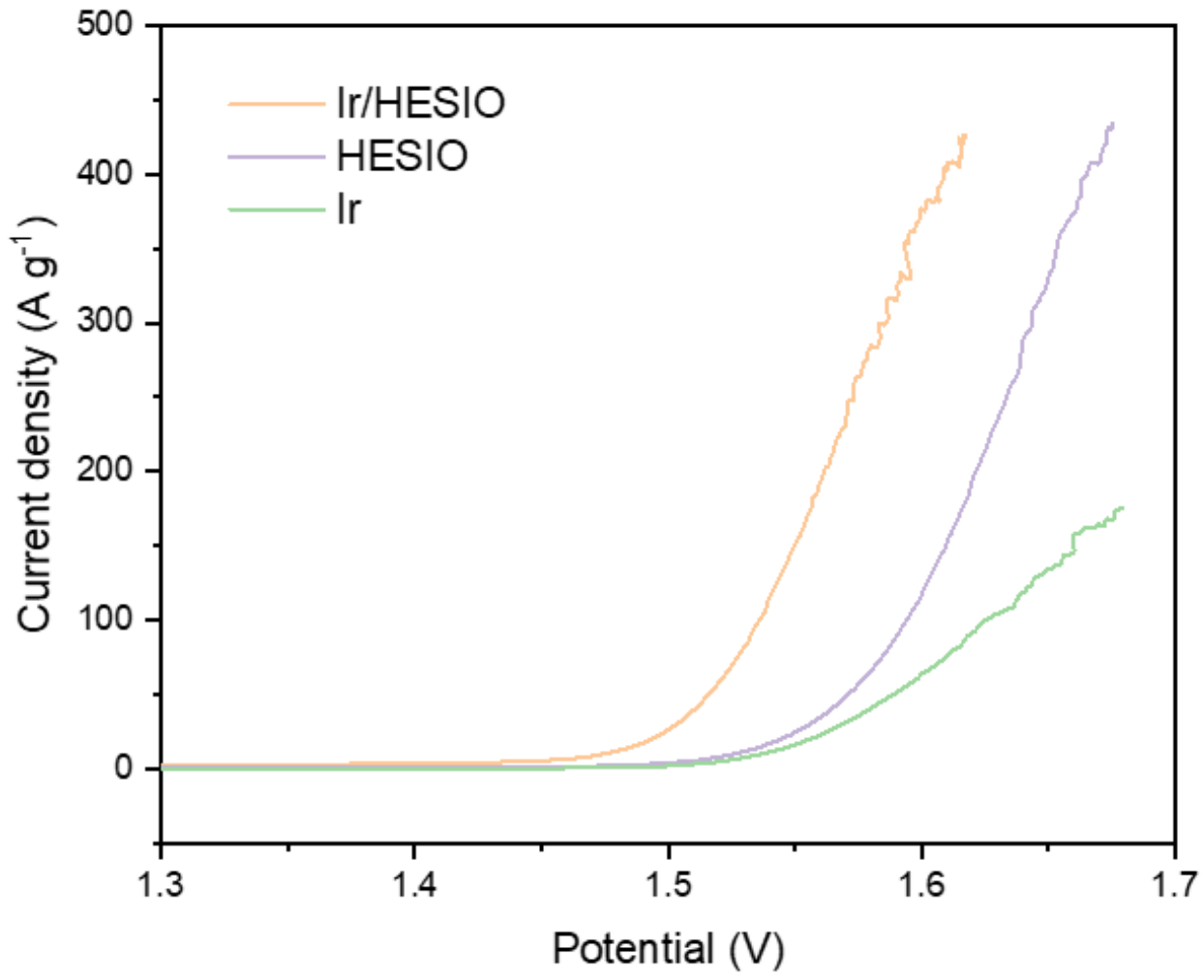

Figure S12 The comparison of mass-specific activities for as-prepared catalysts

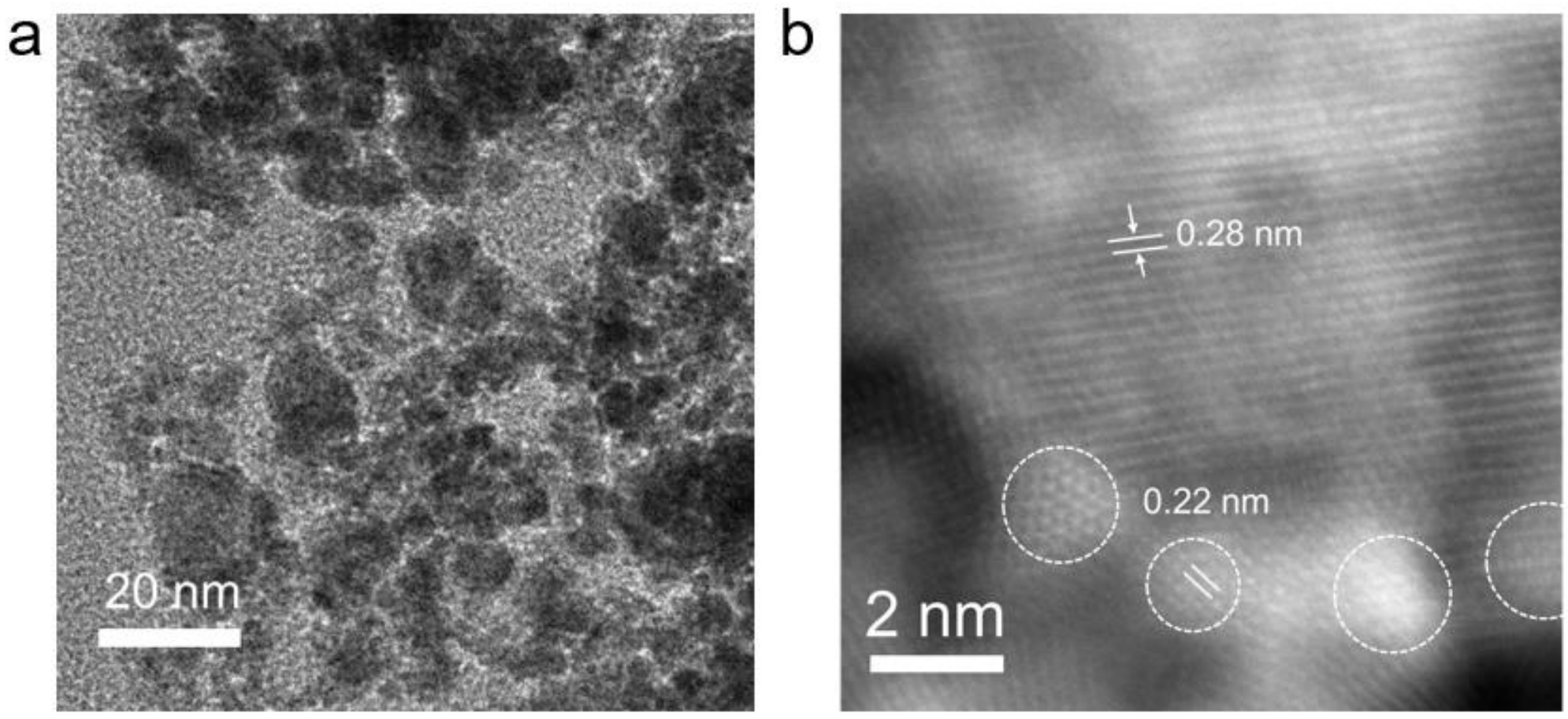

Figure S13 The TEM and HAADF-STEM images for Ir/HESIO after 650 h of CP test

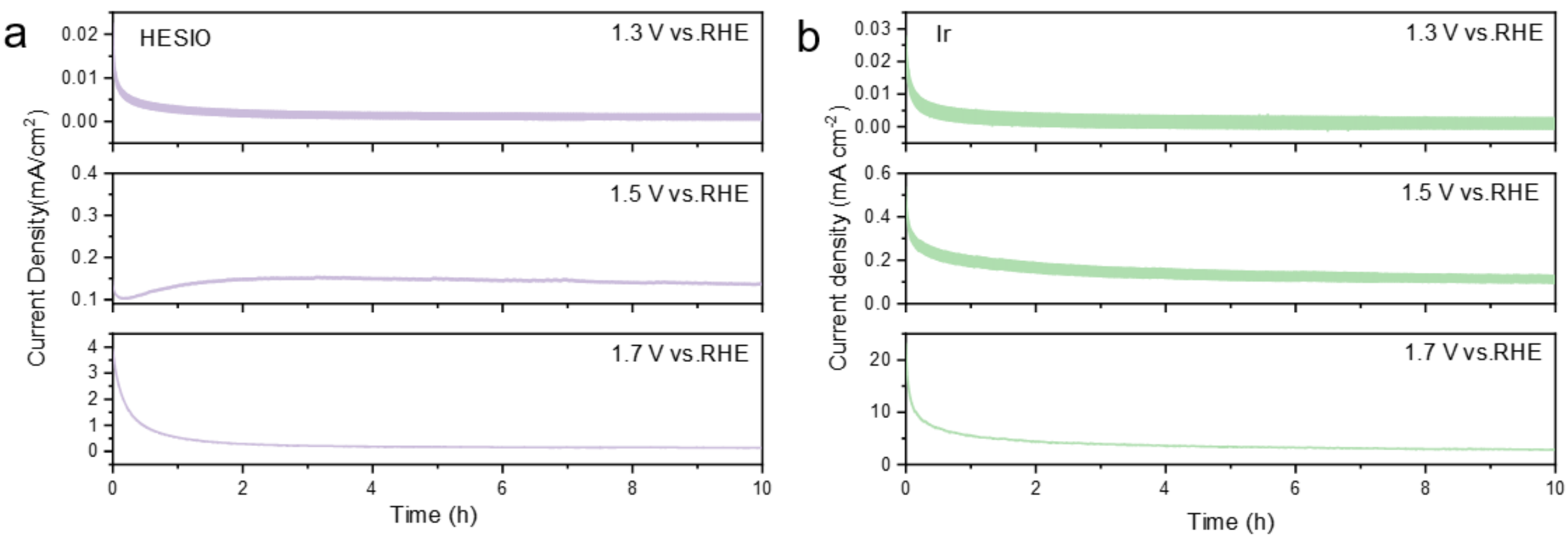

Figure S14 The chronoamperometry curves for **a,** HESIO catalysts and **b,** Ir catalysts in different constant potentials

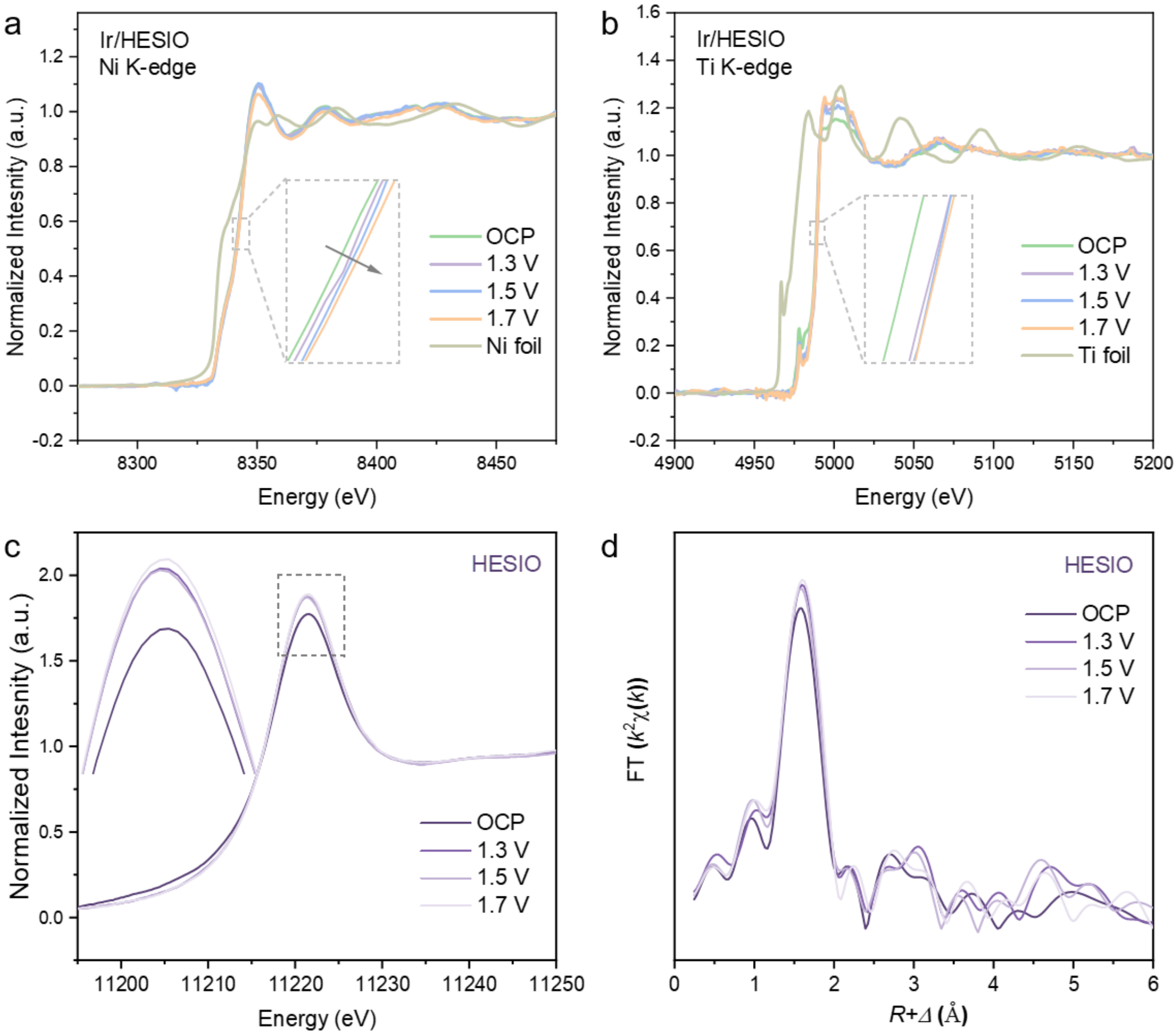

Figure S15 The in-situ XANES spectra in **a,** Ni K-edge and **b,** Ti K-edge for Ir/HESIO at different applied potentials; the in-situ **c,** XANES and **d,** EXAFS results in Ir L_3-edge for HESIO

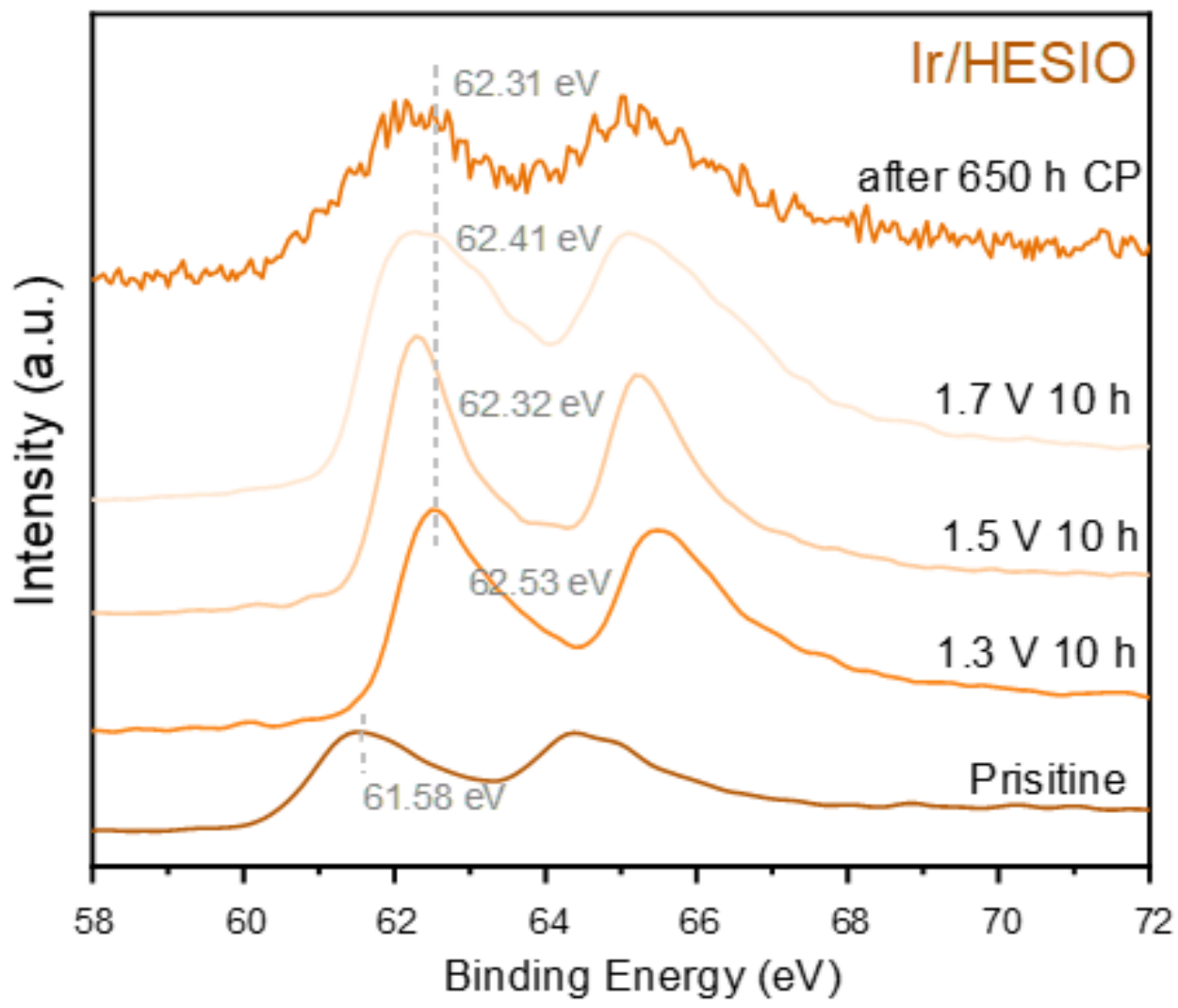

Figure S16 The Ir XPS spectra of Ir/HESIO after 650 h CP test compared with the pristine catalysts and the post-10 h CA catalysts

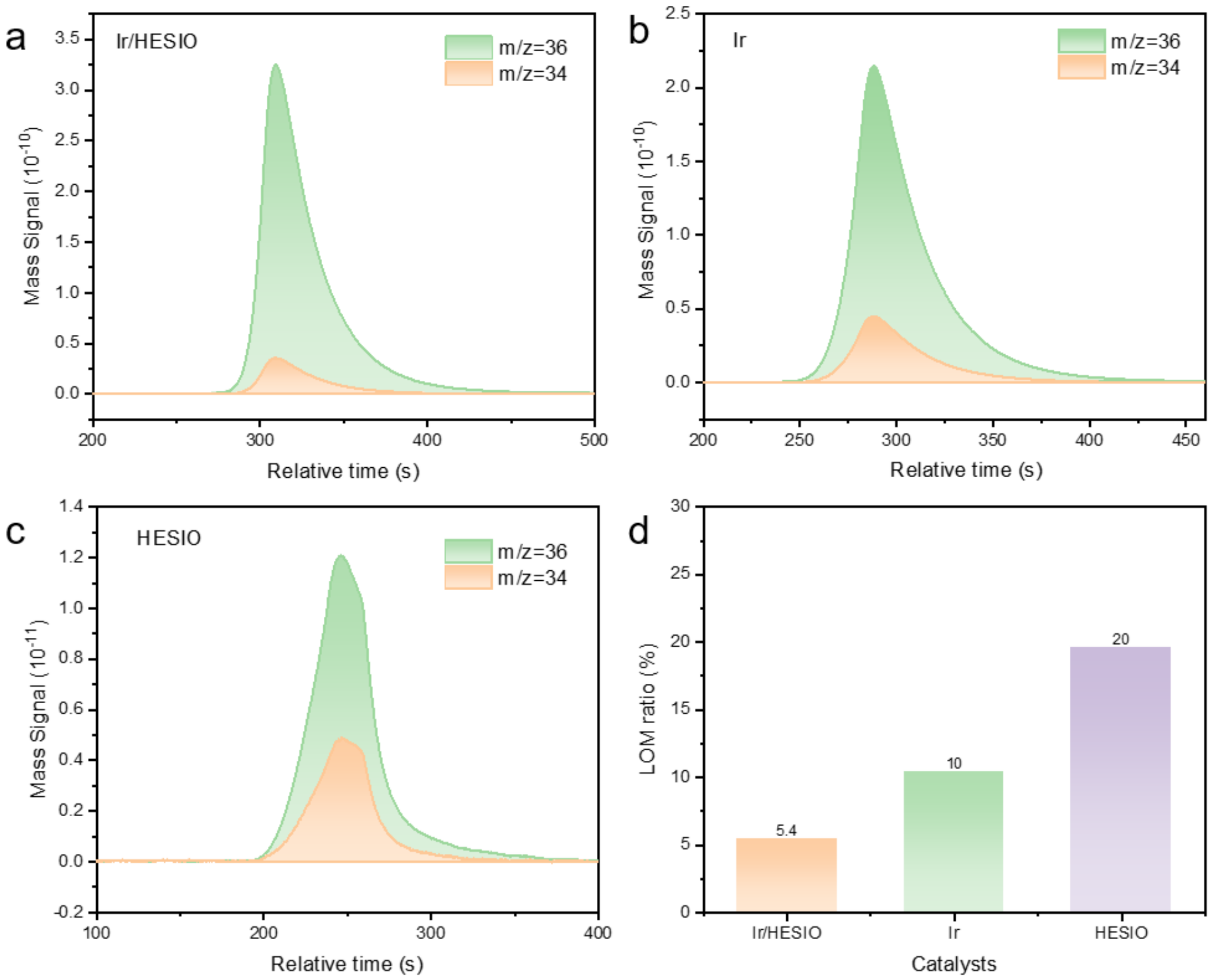

Figure S17 The DEMS signal for **a,** Ir/HESIO; **b,** Ir catalysts and **c,** HESIO; **d,** the LOM ratio for the compared catalysts

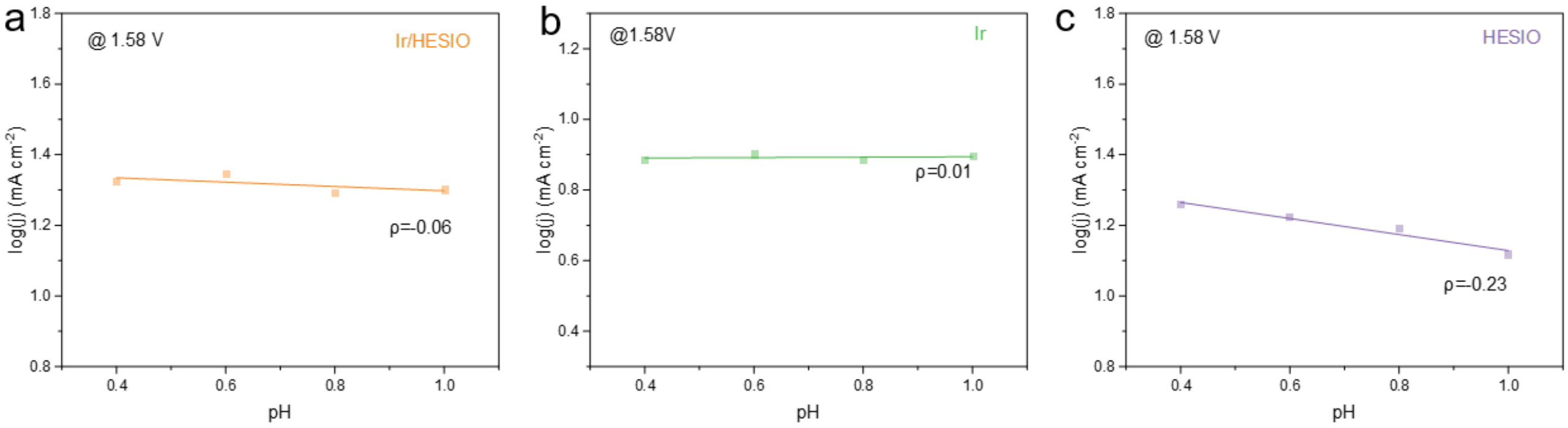

Figure S18 The pH-dependent tests for **a,** Ir/HESIO; **b,** Ir catalyst, and **c,** HESIO

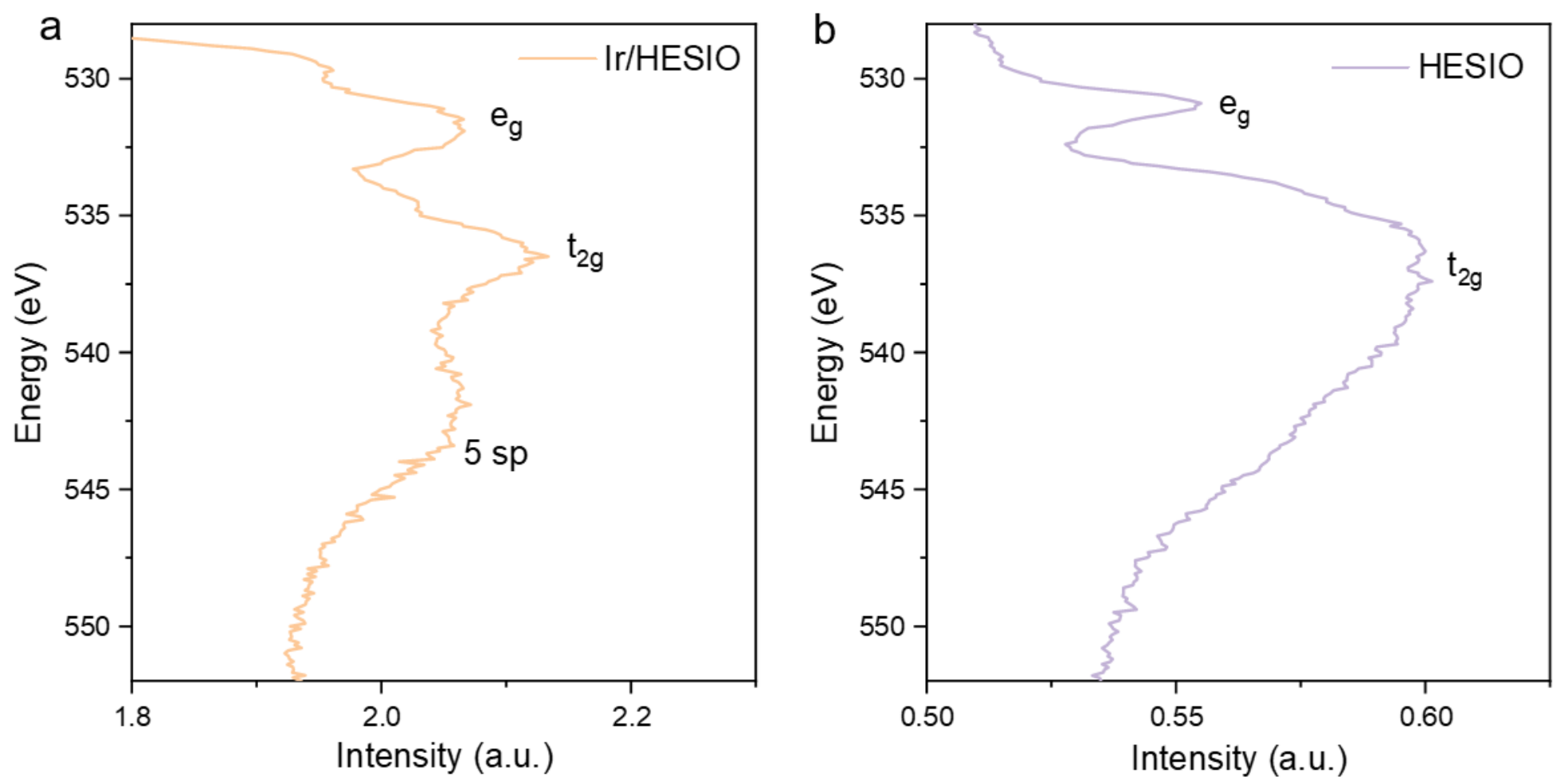

Figure S19 The O K-edge XAS spectra for **a,** Ir/HESIO and **b,** HESIO catalysts

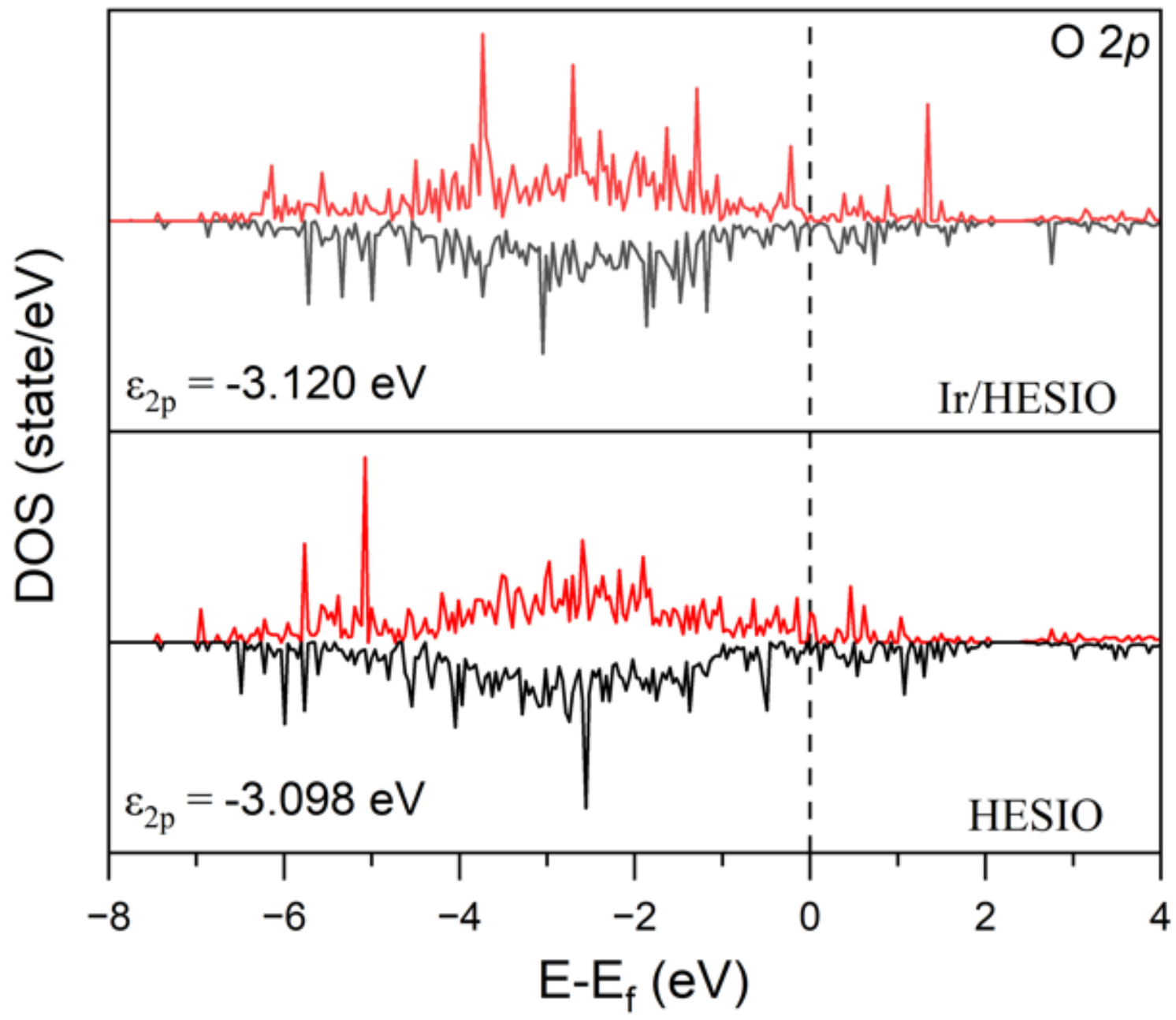

Figure S20 The theoretical O 2p DOS for Ir/HESIO and HESIO models

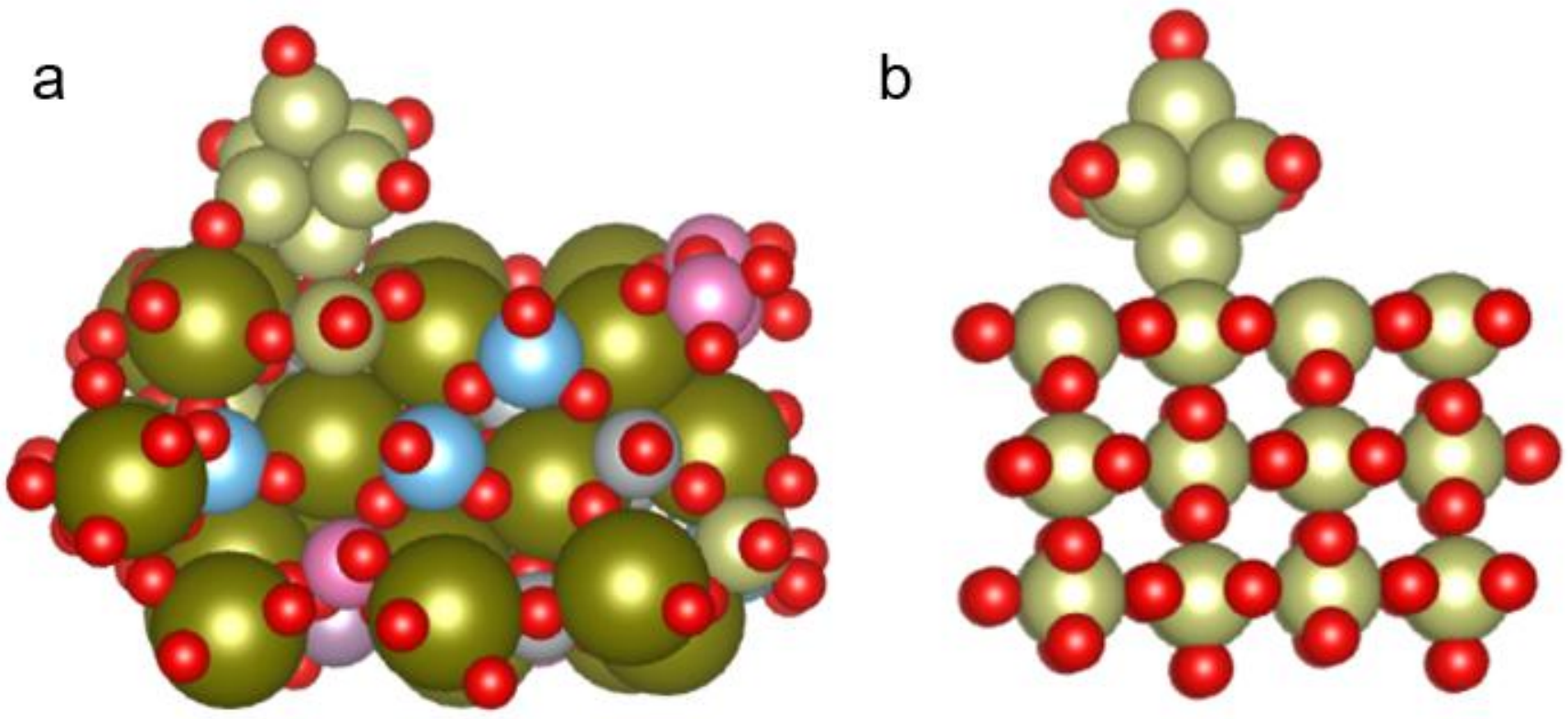

Figure S21 The models of **a,** Ir/HESIO and **b,** Ir/IrO_2 model with 1ML O* cover

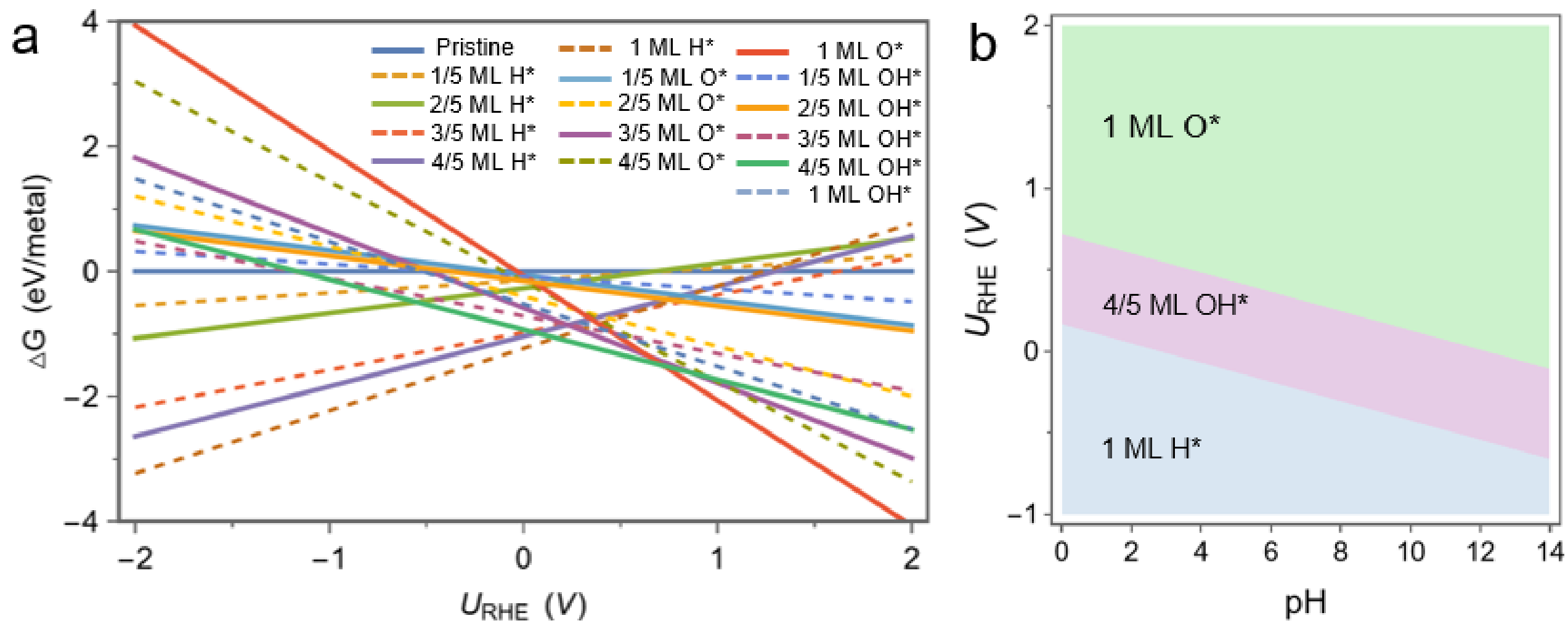

Figure S22 a, Calculated one-dimensional (1D) surface Pourbaix diagram as a function of potential vs. RHE (reversible hydrogen electrode) at 298.15 K; **b,** Corresponding two-dimensional (2D) surface Pourbaix diagram as a function of potential vs. RHE (reversible hydrogen electrode) and pH at 298.15 K for Ir/IrO_2 model

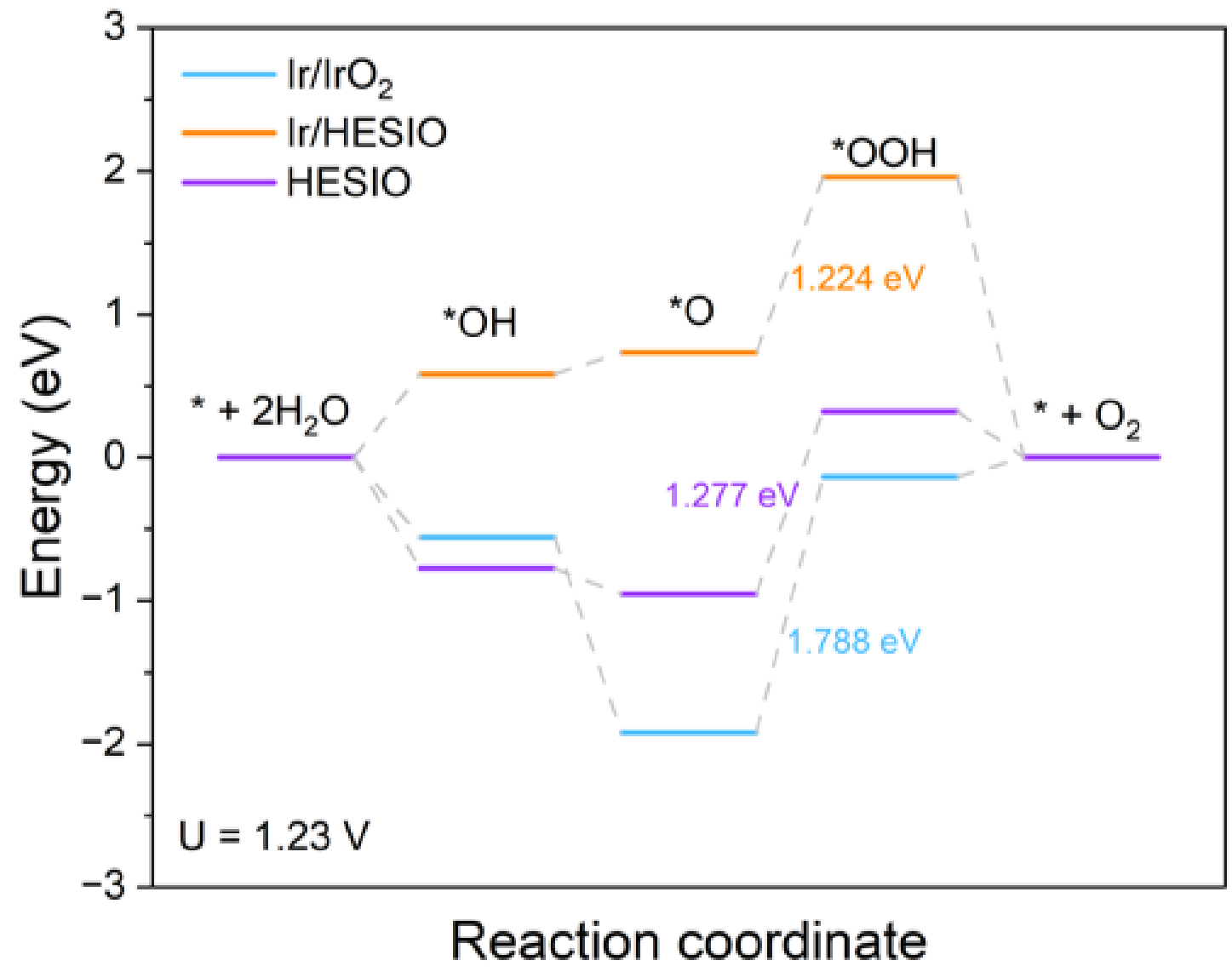

Figure S23 The OER free-energy profiles for the considered models at U = 1.23 V

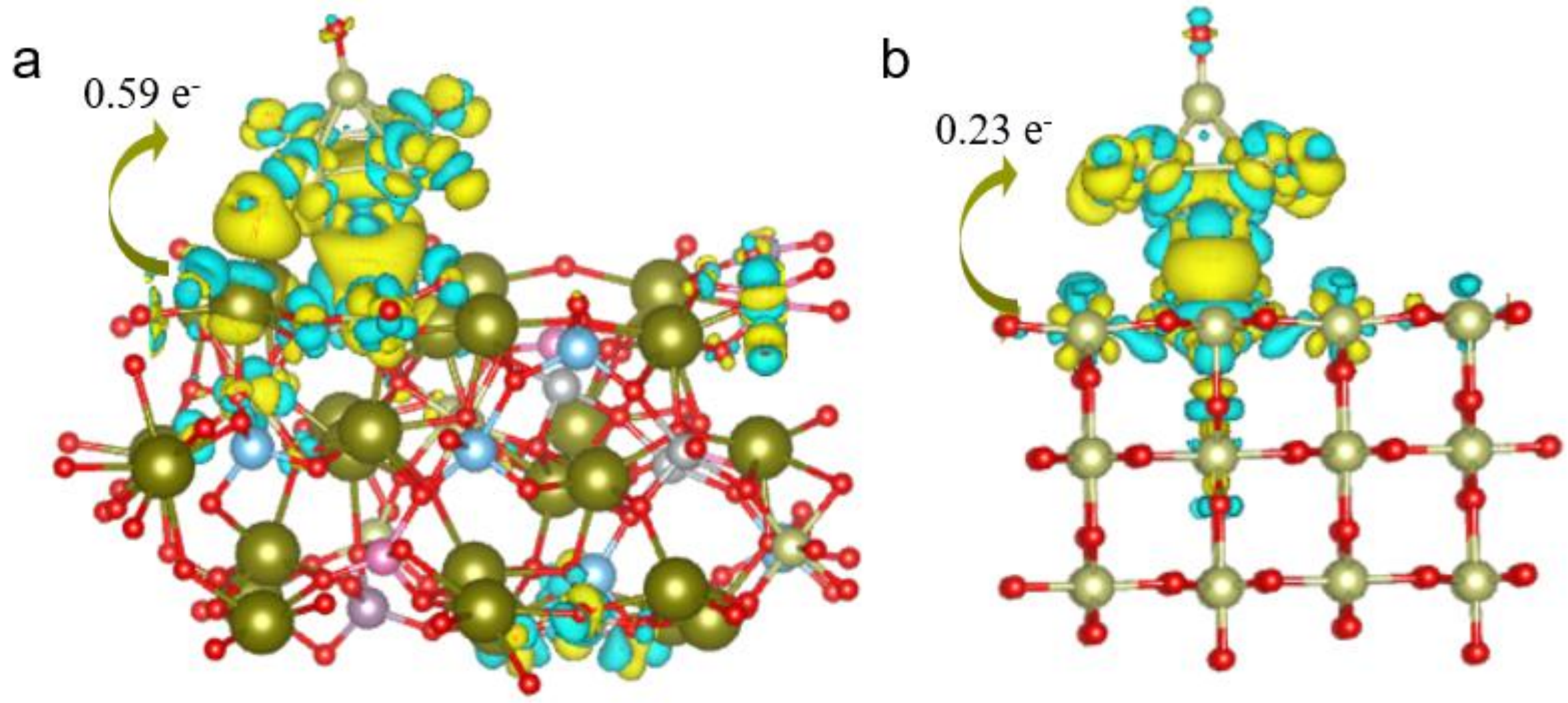

Figure S24 The charge-density-difference and Bader charge analyses of the **a,** Ir/HESIO and **b,** Ir/IrO_2 models

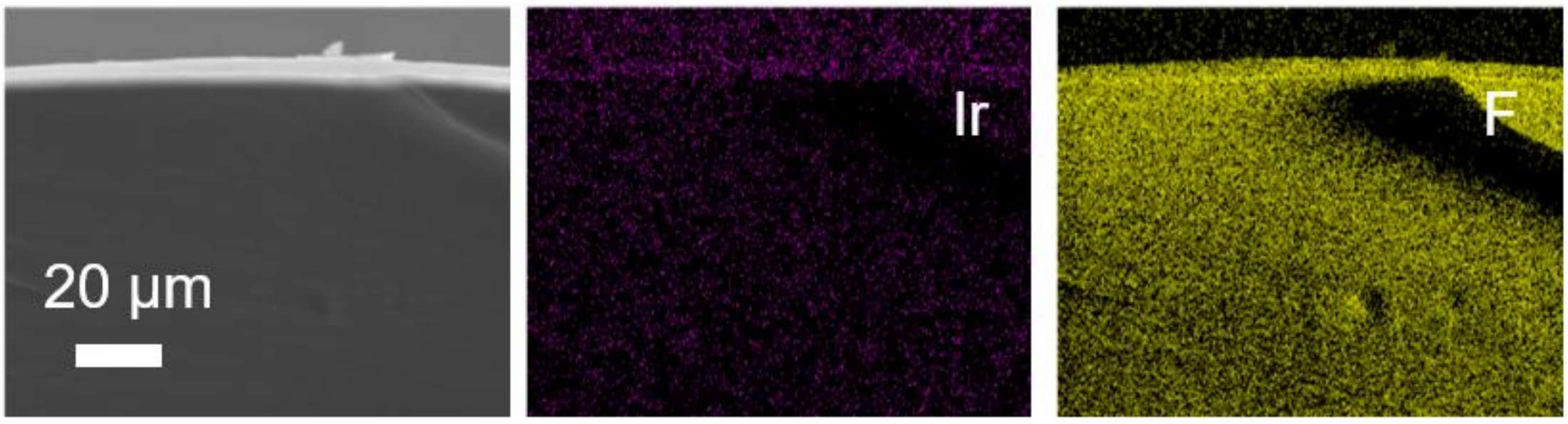

Figure S25 The EDS-mapping of the cross-sectional MEA

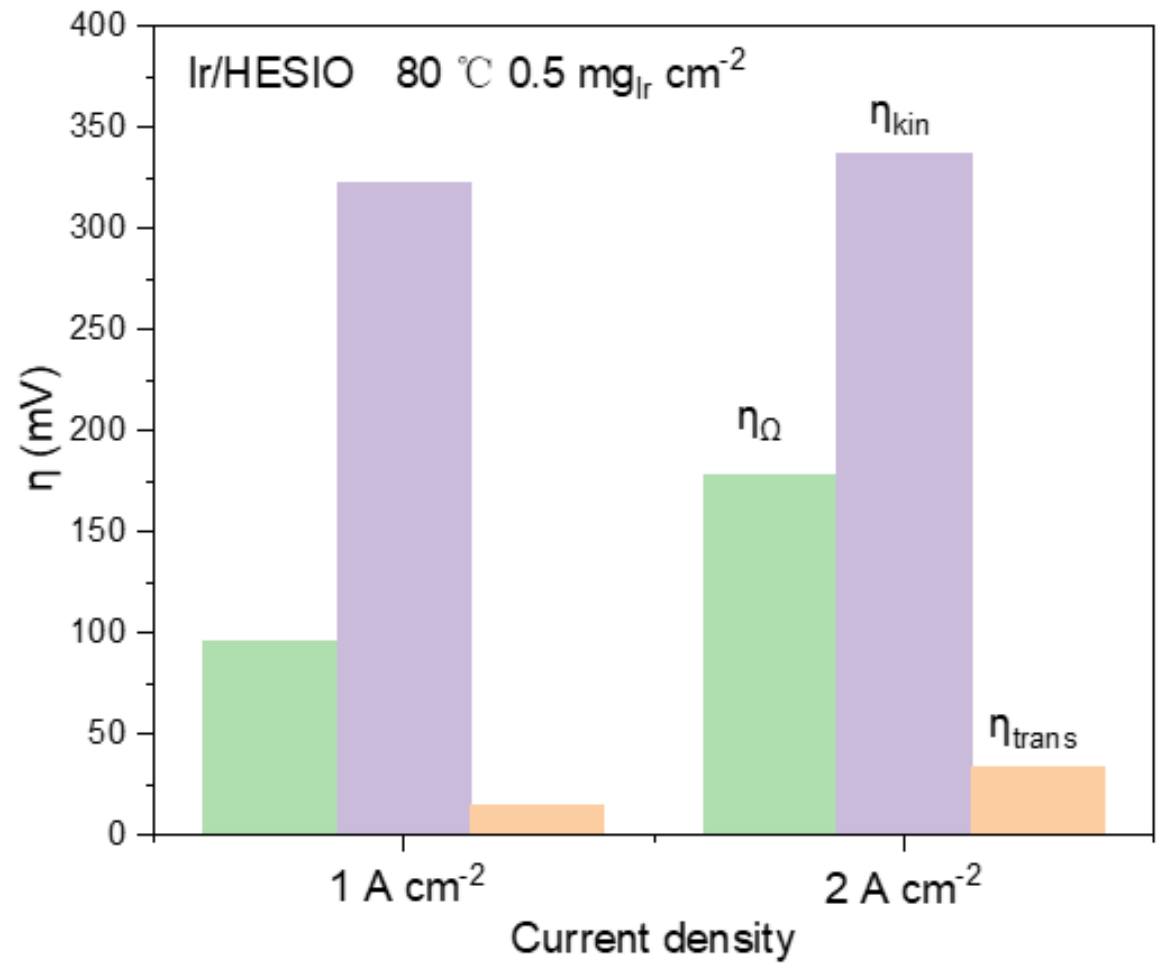

Figure S26 The detailed contribution of each overpotential at different current densities in the MEA fabricated by Ir/HESIO

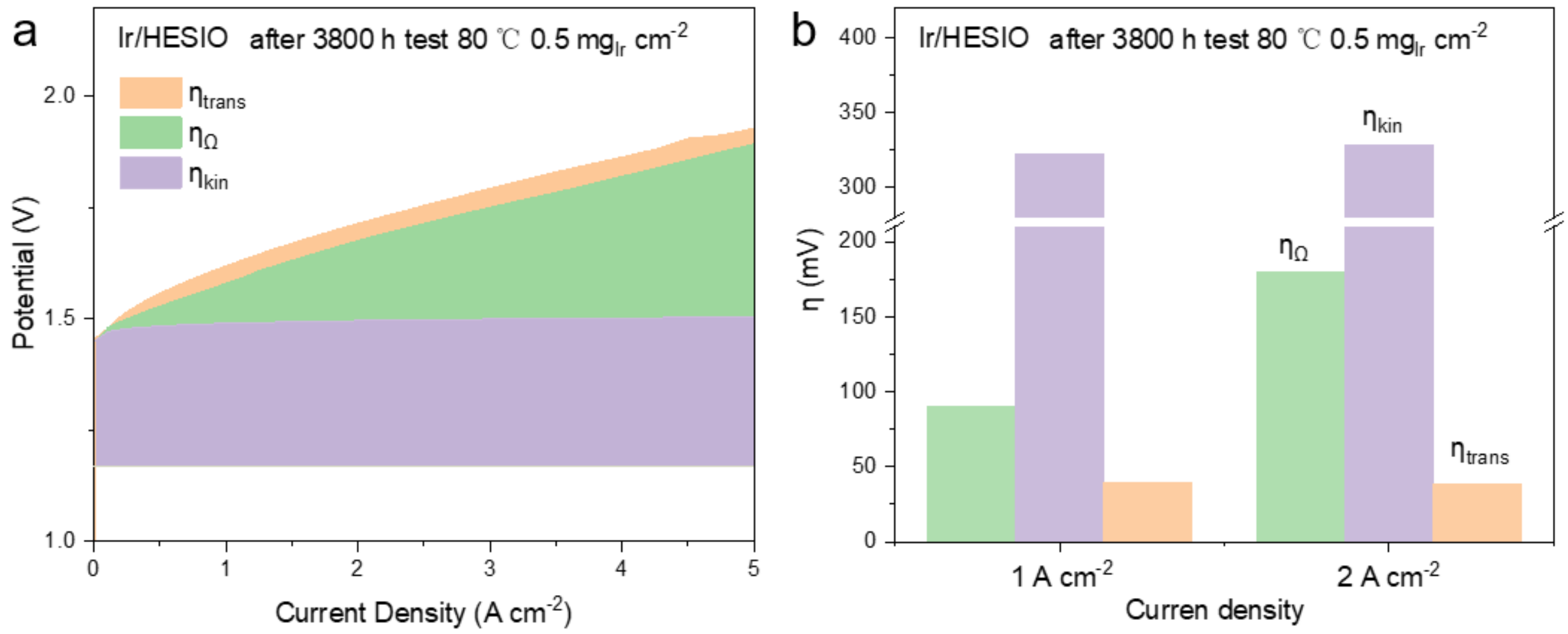

Figure S27 a, the deconvoluted polarization curves of the MEA fabricated by Ir/HESIO after 3800 h test; **b,** the corresponding detailed overpotential contribution at different current densities obtained according to Figure a

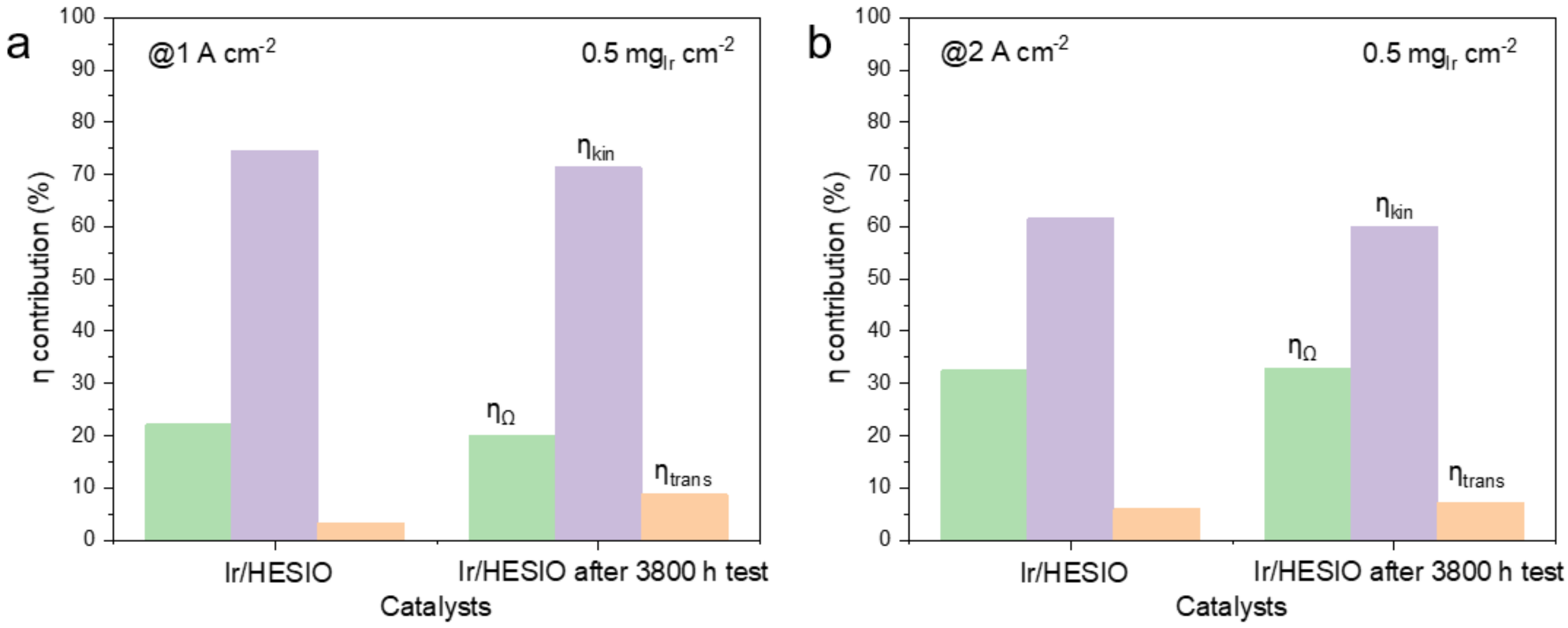

Figure S28 The comparison of the overpotential contributions ratio before and after stability test at a current density of **a,** 1 A cm^{-2}; **b,** 2 A cm^{-2}

Table S3 The comparison of the different overpotential contributions at 2 A cm^{-2} to the total overpotential

**Overpotential contributions**	**η_{Ω}/mV**	**η_{kin}/mV**	**η_{trans}/mV**
**Before the stability test**	178	337	33
**After 3800 h test at 2A cm^{-2}**	179	328	39

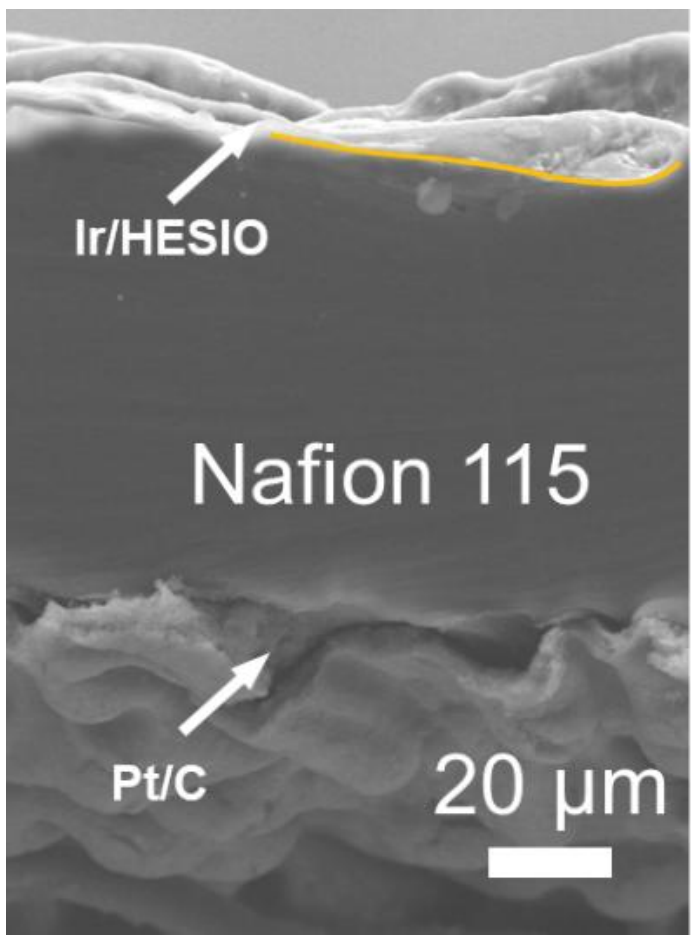

Figure S29 The cross-sectional SEM image of MEA after stability test

Table S4 Comparison of the performance of PEMWE using different anode catalysts

Catalysts	Membrane	Ir loading (mg cm^{-2})	Mass activity (A mg^{-1} @1.7 V)	Cell potential (V, @2 A cm^{-2})	Operation time (h @ A cm^{-2})	Ref
**Ir/HESIO**	Nafion 115	0.525<br>0.284<br>0.156	3.6<br>6.2<br>8.8	1.71<br>1.73<br>1.79	3827@2; 3200@3<br>——<br>——	This work
**$Ca_{1.6}K_{0.4}IrO_{4-x}$**	Nafion 115	0.7	~1.9	1.75	520@1	12
**IrO_x/WO_xNW**	Nafion 115	0.33	~4.5	1.79	200@1	13
**Ir/P_{120}-NTO**	Nafion 115	0.5	~2.07	~1.82	500@1	14
**IrCo@TiN**	Nafion 117	0.2	~2.98	~1.95	200@1	15
**IrO_2/S**	Nafion 115	0.3	~4.81	1.769	1000@1	16
**Ir/MnO_x**	Nafion 117	0.2	~6.65	~1.8	300@1	17
**$Ir@Ga_2O_3$**	Nafion 212	——	——	1.76	1000@1	18
**$Ir/IrO_x(OH)_y\cdot(H_2O)_n$**	Nafion 115	——	——	~2	1200@1	19
**$IrO_2@TaO_x@TaB$**	Nafion 115	0.26	~5.76	~1.75	1500@2	20
**Ir-3 MA**	Nafion 212	0.5	2.7	1.79	400@2	21
**KIr_4O_8 Nanowires**	Nafion 115	2.0	~0.57	1.80	1230@1	22
**$IrOx/\gamma$-MnO_2**	Nafion 212@50℃	0.28	~3.03	~2.03	1000@0.25<br>400@1	23
**$IrO_2(101)$ monolayer**	Nafion 115	1.5	1.33	1.70	10000@1.5	24
**RIE-Ir/CeO_x**	Nafion 115 or 212	0.3	~9.67	~1.60	6000@1-8	25

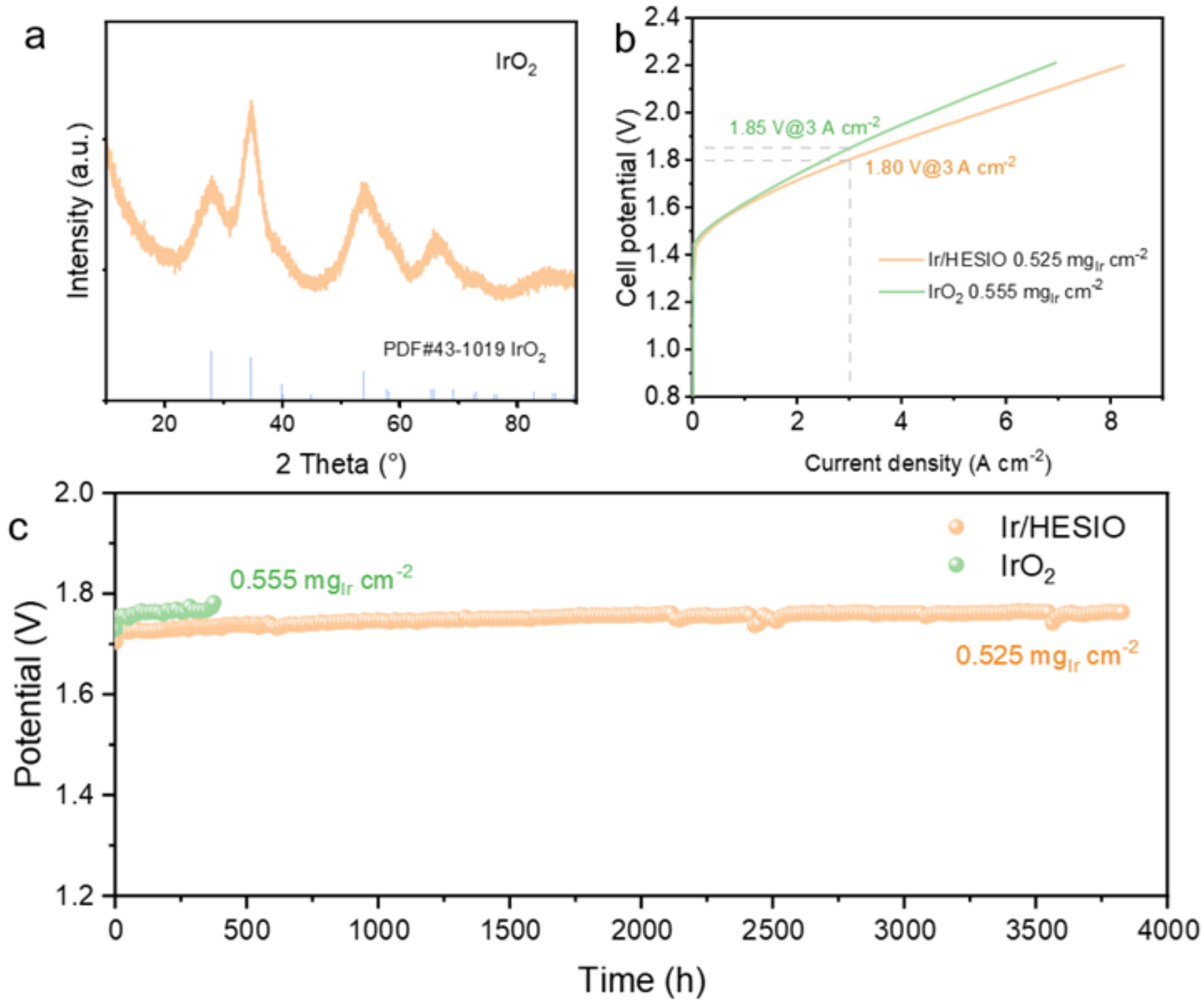

Figure S30 a, The XRD pattern of the IrO_2 sample; **b,** The comparison of polar curves for Ir/HESIO and IrO_2 in a PEMWE single-cell; **c,** The stability test at a constant current density of 2 A cm^{-2} in a PEMWE single-cell

References:

(1) Yang, L.; Yu, G.; Ai, X.; Yan, W.; Duan, H.; Chen, W.; Li, X.; Wang, T.; Zhang, C.; Huang, X.; Chen, J. S.; Zou, X., Efficient Oxygen Evolution Electrocatalysis in Acid by a Perovskite with Face-sharing IrO_6 Octahedral Dimers. *Nat. Commun.* **2018,** *9* (1), 5236.

(2) Chen, H.; Shi, L.; Liang, X.; Wang, L.; Asefa, T.; Zou, X., Optimization of Active Sites via Crystal Phase, Composition, and Morphology for Efficient Low-Iridium Oxygen Evolution Catalysts. *Angew. Chem. Int. Ed.* **2020,** *59* (44), 19654-19658.

(3) Kresse G; Furthmu, È. J., Efficient Iterative Schemes for Ab initio Total-energy Calculations Using a Plane-wave Basis Set. *Phys. Rev. B* **1996,** *54* (16), 11169-11186.

(4) Blöchl, P. E., Projector Augmented-Wave Method. *Phys. Rev. B* **1994,** *50* (24), 17953-17979.

(5) John P. Perdew, K. B., Matthias Ernzerhof, Generalized Gradient Approximation Made Simple. *Phys. Rev. Lett.* **1996,** *77* (18), 3865-3868.

(6) B. Hammer; L. B. Hansen; Nørskov, J. K., Improved Adsorption Energetics within Density-functional Theory Using Revised Perdew-Burke-Ernzerhof Functionals. *Phys. Rev. B* **1999,** *59* (11), 7413-7421.

(7) Monkhorst, H. J.; Pack, J. D., Special Points for Brillouin-zone Integrations. *Phys. Rev. B* **1976,** *13* (12), 5188-5192.

(8) S. L. Dudarev, G. A. B., S. Y. Savrasov, C. J. Humphreys, A. P. Sutton, Electron-energy-loss spectra and the structural stability of nickel oxide: An LSDA U study. *Phys. Rev. B* **1998,** *57*, 1505-1509.

(9) J. K. Nørskov, J. R., A. Logadottir, L. Lindqvist, J. R. Kitchin, T. Bligaard, H. Jónsson, Origin of the Overpotential for Oxygen Reduction at a Fuel-Cell CathodeClick to copy article link. *J. Phys. Chem. B* **2004,** *108* (46), 17886-17892.

(10) Gauthier, J. A.; Dickens, C. F.; Chen, L. D.; Doyle, A. D.; Nørskov, J. K., Solvation Effects for Oxygen Evolution Reaction Catalysis on IrO_2(110). *J. Phys. Chem. C* **2017,** *121* (21), 11455-11463.

(11) Liu, H.; Zhang, D.; Holmes, S. M.; D'Agostino, C.; Li, H., Origin of the superior oxygen reduction activity of zirconium nitride in alkaline media. *Chem. Sci.* **2023,** *14* (34), 9000-9009.

(12) Liang, H.; Cheng, H.; Zhao, B.; Li, Y.; Shi, C.; Zhang, J.; Ding, Y.; Cui, H.; Hu, H., Engineering a Synergistic Mechanism of K-O-Ir Bonding and Oxygen Vacancies for Durable and Accelerated Oxygen Evolution in Layered Ir-based Perovskites. *Appl Catal B: Environ* **2026,** *382*, 125962.

(13) Chueh, L.; Chien, Y.; Hsu, Y.; Wang, Z.; Tsai, D.-H.; Wu, C.-M.; Huang, S.-C.; Chen, H.-J.; Chen, H.-Y.; Tsai, M.-H.; Wang, C.-H.; Yang, C.-C.; Pan, Y.-T., Suppressing Iridium Over-oxidation via Catalyst-support Interactions on Tungsten oxide Nanowires Revealed by in-situ XPS for Durable, Low-loaded PEM Water Electrolysis. *Appl Catal B: Environ* **2026,** *382*, 125919.

(14) Zhu, Y.; Guo, F.; Wei, Q.; Lai, F.; Chen, R.; Guo, J.; Gong, M.; Zhang, S.; Wang, Z.; Zhong, J.; He, G.; Cheng, N., Engineering the Metal/Oxide Interfacial O-Filling Effect to Tailor Oxygen Spillover for Efficient Acidic Water Oxidation. *Adv. Funct. Mater.* **2025,** *35* (22), 2421354.

(15) Yang, Y.; Chen, D.; Wang, L.; Hu, S.; Pei, P.; Xu, X., TiN Anchored Ultrafine IrCo Alloy with Strong Electronic Coupling Effect as Highly-efficient OER Electrocatalyst for Proton Exchange Membrane Electrolyzers. *Chem. Eng. J.* **2025,** *516*, 164061.

(16) Yang, C.; Zhu, Y.; Zhang, F.; Yao, L.; Chen, Y.; Lu, T.; Li, Q.; Li, J.; Wang, G.; Cheng, Q.; Yang,

H., Sulfur-Doped IrO_2 Enable Pathway Switch to Lattice Oxygen Mechanism with Enhanced Stability for Low Iridium PEM Water Electrolysis. *Adv. Mater.* **2025,** *37* (38), 2507560.

(17) Wang, D.; Lin, F.; Luo, H.; Zhou, J.; Zhang, W.; Li, L.; Wei, Y.; Zhang, Q.; Gu, L.; Wang, Y.; Luo, M.; Lv, F.; Guo, S., Ir-O-Mn Embedded in Porous Nanosheets Enhances Charge Transfer in Low-Iridium PEM Electrolyzers. *Nat. Commun.* **2025,** *16* (1), 181.

(18) Qian, Y.; Guo, Y.; Yang, Z.; Luo, Z.; Zhang, L.; Zhang, Q.; He, C.; Zhang, H.; Sun, X.; Ren, X., Bias-Induced Ga-O-Ir Interface Breaks the Limits of Adsorption-Energy Scaling Relationships for High-Performing Proton Exchange Membrane Electrolyzers. *Angew. Chem. Int. Edit.* **2025,** *64* (10), e202419352.

(19) Luo, Z.; Qian, Y.; Yang, Z.; Zhang, L.; Zhang, Q.; He, C.; Ren, X., Water-Hydroxide Trapping in Hollandite-Type Iridium Oxide Enables Efficient Proton Exchange Membrane Water Electrolysis. *Adv. Funct. Mater.* **2025,** *35* (25), 2500044.

(20) Wang, Y.; Zhao, Z.; Liang, X.; Zhao, X.; Wang, X.; Jana, S.; Wu, Y. A.; Zou, Y.; Li, L.; Chen, H.; Zou, X., Supported IrO_2 Nanocatalyst with Multilayered Structure for Proton Exchange Membrane Water Electrolysis. *Adv. Mater.* **2024,** *36* (39), 2407717.

(21) Liang, J.; Fu, C.; Hwang, S.; Dun, C.; Luo, L.; Shadike, Z.; Shen, S.; Zhang, J.; Xu, H.; Wu, G., Constructing Highly Porous Low Iridium Anode Catalysts Via Dealloying for Proton Exchange Membrane Water Electrolyzers. *Adv. Mater.* **2024,** *37* (4), 2409386

(22) Li, Z.; Li, X.; Wang, M.; Wang, Q.; Wei, P.; Jana, S.; Liao, Z.; Yu, J.; Lu, F.; Liu, T.; Wang, G., KIr_4O_8 Nanowires with Rich Hydroxyl Promote Oxygen Evolution Reaction in Proton Exchange Membrane Water Electrolyzer. *Adv. Mater.* **2024,** *36* (30), 2402643.

(23) Ko, W.; Shim, J.; Ahn, H.; Kwon, H. J.; Lee, K.; Jung, Y.; Antink, W. H.; Lee, C. W.; Heo, S.; Lee, S.; Jang, J.; Kim, J.; Lee, H. S.; Cho, S.-P.; Lee, B.-H.; Kim, M.; Sung, Y.-E.; Hyeon, T., Controlled Structural Activation of Iridium Single Atom Catalyst for High-Performance Proton Exchange Membrane Water Electrolysis. *J. Am. Chem. Soc.* **2025,** *147* (3), 2369-2379.

(24) Yang, D.; Zhang, C.; Qin, Y.; Yue, Y.; Liu, Y.; Shi, X.; Hua, K.; An, X.; Jin, L.; Zhang, Y.; Zuo, S.; Tan, A.; Liu, J., Single-faceted IrO_2 Monolayer Enabling High-performing Proton Exchange Membrane Water Electrolysis beyond 10,000 h Stability at 1.5 A cm^{-2}. *Nat. Commun.* **2025,** *16* (1), 7236.

(25) Shi W.; Shen T.; Xing C.; Sun K.; Yan Q.; Niu W.; Yang X.; Li J.; Wei C.; Wang R.; Fu S.; Yang Y.; Xue L.; Chen J.; Cui S.; Hu X.; Xie K.; Xu X.; Duan S.; Xu Y.; Zhang, B., Ultrastable Supported Oxygen Evolution Electrocatalyst Formed by Ripening-induced Embedding. *Science* **2025,** *387* (6735), 791-796.
